# Improved Dual-Modality Bioequivalence Evaluation of Topical Formulations Within Human Skin Using Stimulated Raman Scattering Microscopy

**DOI:** 10.3390/pharmaceutics17091193

**Published:** 2025-09-13

**Authors:** Dandan Tu, Nick-Sidney Lemberger, Kristin Wallmeier, Jackson Riseman, Benjamin A. Kuzma, Yuxiao Wei, Ting Chean Khoo, Elena Rantou, Priyanka Ghosh, Markham C. Luke, Sam G. Raney, Carsten Fallnich, Conor L. Evans

**Affiliations:** 1 Wellman Center for Photomedicine, Massachusetts General Hospital, Harvard Medical School, CNY149-3, 13th St, Charlestown, MA 02129, USA; datu@mgh.harvard.edu (D.T.); ben_kuzma@vrtx.com (B.A.K.); tkhoo@mgh.harvard.edu (T.C.K.); 2 Institute of Applied Physics, University of Münster, 48149 Münster, Germanyfallnich@uni-muenster.de (C.F.); 3 Office of Biostatistics, Office of Translational Sciences, Center for Drug Evaluation and Research, U.S. Food and Drug Administration, Silver Spring, MD 20993, USA; 4 Office of Research and Standards, Office of Generic Drugs, Center for Drug Evaluation and Research, U.S. Food and Drug Administration, Silver Spring, MD 20993, USA

**Keywords:** topical product, bioequivalence, stimulated Raman microscope, cutaneous pharmacokinetics, light intensity normalization, multi-modality

## Abstract

**Background:** The use of optical microscopic techniques has gained increasing attention in recent years for studying the bioavailability (BA) and bioequivalence (BE) of topical drugs. Stimulated Raman scattering (SRS), one type of optical imaging technique, probes chemical-specific information and has excellent spatiotemporal resolution. It uses intrinsic molecular vibrational signatures, and therefore, labeling samples or other treatments is unnecessary to track a molecule. Because of its unique advantages, studies have used SRS for BA evaluations and, more recently, for BE evaluations. In BE evaluation, low data variance within a treatment group is important to ensure sensitivity and specificity in comparing treatment groups. **Methods:** When measuring forward-direction SRS signals transmitted through skin, the signal intensity is susceptible to variance due to several factors, such as the microscope system’s performance, the different optical features of topical drug products, and the heterogeneity of skin in transmitting light. This work closely investigated the effects of these factors on an SRS signal and developed solutions to reduce their effects on the data variance. Specifically, we constructed a method using a dual-modality detector built in-house, which simultaneously measured both the SRS signal and total light transmission synchronized in time and co-registered in space. **Results:** We developed equations to normalize SRS signals using the transmission intensity, and the results demonstrated a clear improvement in the SRS signal via a reduction in the signal variance (up to a 9.46% CV value decrease) that is otherwise caused by various factors associated with the use of topical drugs and the composition of the skin. We carried out an exploratory BE study using tretinoin-containing topical products and observed improvements in BE assessment with the developed method (could achieve a reduction of 0.11 in the CI value). **Conclusions:** This work has led to a better understanding of the factors that affect SRS imaging and has provided an effective method to compensate for these factors in BE assessments. This is a critical initial effort for better practical implementation of SRS in cutaneous pharmacokinetics (cPKs) studies of topical drugs.

## 1. Introduction

Optical microscopic techniques have been widely used in imaging skin and dermatological drugs [1,2]. Optical microscopic techniques enable 2D/3D spatial imaging, which is critical to obtain microscale information on the skin and show drugs’ distribution in the skin’s compartments. Vibrational microscopic imaging provides an effective way to probe a sample’s intrinsic molecular vibrational signatures, which are chemically specific [1]. Samples can be imaged without chemical treatment (e.g., fixation, labeling), thus preventing perturbation of their original morphology and constituents. Moreover, the capability to image skin with drugs applied to it under non-perturbed conditions makes continual time-lapse imaging possible.

Two pharmaceutical products are considered bioequivalent when (among other criteria) there is no significant difference in the rate at and extent to which the active pharmaceutical ingredient (API) becomes available at the site(s) of drug action [3]. Bioequivalence (BE) assessment is critical in developing a generic version of a reference listed drug (RLD) or to support post-approval changes in an approved drug product [4]. Cutaneous pharmacokinetics (cPKs) approaches have been considered accurate, sensitive, and reproducible for determining the bioavailability (BA) of a dermatological drug or when evaluating the BE between products [4].

Vibrational microscopic imaging, including infrared (IR) imaging [5,6], spontaneous Raman imaging [6,7,8,9,10], and coherent Raman imaging [11,12,13], have been used to characterize the extent and rate of dermatological drug permeation and to assess their BA. Although a spectrometer alone is capable of measuring signals, its ability to relate a signal to the microstructure is lacking. To retrieve spatial information, measurements from spectrometers can be combined with data obtained using methods such as tape stripping to characterize the depth and distribution indirectly [6,14]. In contrast, microscopic imaging, which generally combines the use of a spectrometer with a microscope, is capable of measuring signals corresponding to different spatial locations. An IR imaging microscope has been used in imaging the spatial distribution of drugs [5]. However, the low lateral spatial resolution of IR imaging makes the resolution of signals and the fine features of skin microstructures challenging [15]. A confocal Raman microscope measures the spontaneous Raman scattering signal and has high lateral spatial resolution, capable of resolving features at the cellular and sub-cellular levels [15]. Lab-built, custom confocal Raman microscopes, as well as commercial systems (e.g., the WiTec alpha 500 and the River Diagnostics gen2 SCA device, from Wissenschaftliche Instrumente und Technologie GmbH, Ulm, Germany), have been used extensively in studying the pharmacokinetics of topical drug products applied to the skin [7,8,9,10,16,17]. However, their long acquisition time (milliseconds to seconds) per pixel means that it takes hours to days to collect a high-spatial-resolution image. A trade-off between the image’s spatial resolution and temporal resolution is unavoidable when using spontaneous Raman imaging.

Stimulated Raman scattering (SRS), one modality of coherent Raman scattering, probes the intrinsic molecular vibrational signatures and is capable of resolving features at the cellular and sub-cellular levels. SRS not only has good spatial resolution but also has good temporal resolution for video-rate imaging. In addition, SRS is insensitive to skin’s autofluorescence, which is a limitation of spontaneous Raman imaging of skin. Because of its unique advantages, SRS has been used extensively in cPK studies of topical formulations. It has been used in studying the permeation of a topical ibuprofen formulation within porcine skin and the associated morphological changes [18] and showing the depth distribution of 4-cyanophenol [19], lidocaine, and loxoprofen [20] in the skin. SRS has also been used to image the distribution of *trans*-retinol [21], ruxolitinib [11,12,22], deuterated betamethasone dipropionate [11], tazarotene [23], 4-cyanophenol [24], and zinc pyrithione and climbazole [25] in different skin microstructures. More recently, an SRS-based method has been used in a study to assess the BE of dermatological drugs containing tazarotene [26].

The majority of these studies measured forward-direction SRS signals transmitted through ex vivo skin samples [11,12,18,19,20,23,24,25,26]. Forward-direction SRS refers to measuring the SRS signal when the light travels in the same direction as the incident light. To measure the signal, a detector is positioned on the opposite side of the incident laser beams to measure the light transmitted through the skin. The measured SRS intensity is dependent on the skin’s thickness and pigmentation variance, largely because keratin in the epidermis and collagen in the dermis are highly scattering, and melanin pigmentation exhibits significant absorption of light below 1100 nm [27,28,29,30,31]. Besides those of skin, the optical features of applied formulations also alter the measured light intensity. There are a variety of topical dosage forms, ranging from simple liquids (e.g., solutions) to semisolids (e.g., gels and creams) and solids (e.g., powders). The distinct optical features of transparent solutions and opaque creams result in differences in light transmission. Moreover, there can be different microstructures in the same type of dosage form (e.g., different droplet sizes in different creams) [32]; the different droplet sizes in creams can change the extent to which light scatters according to Mie scattering theory. In addition, the thickness of the formulation is another factor that impacts the extent of light attenuation, thus adding more variance to the measured transmitted SRS signal. Assessing the variance caused by these factors and reducing their impact is critical for the reliable application of SRS in cPK studies. However, these factors have not been well considered in previous studies, and there is currently a need for a method to reduce this variance in SRS signals.

Tretinoin (all-trans retinoic acid) is an active ingredient with keratolytic and anti-inflammatory effects. It has been used to treat acne vulgaris, psoriasis, and photoaging [33,34]. Considering the potential teratogenicity of systemically available tretinoin [35,36], topical administration of tretinoin is widely used for treatment of skin diseases. Currently, the cPKs of tretinoin are challenging to evaluate in human skin, whether studied using Franz cells [33,34,37] or tape stripping [36,38], and they are quantified using HPLC [33,34,37]. It is challenging to retrieve spatial distribution information using the Franz cell method. The tape stripping method allows for sampling at different depths; however, the procedure perturbs the sampling area, which could constitute a barrier to repetitive measurements. In contrast to these methods, SRS imaging has the potential to provide 3D spatial imaging, with high temporal resolution and the ability to track active pharmaceutical ingredients (APIs) under non-perturbed conditions during continual time-lapse imaging. The application of SRS imaging in studying the BA or BE of tretinoin-containing topical products could help us understand the BA of the drug in these products, thereby facilitating product development.

In this study, a dual-modality detector, which simultaneously measures SRS and near-infrared light (NIR) transmission, was used to image an API in topical formulations on ex vivo human skin. The NIR intensity and SRS signal were measured simultaneously with the same excitation light, enabling optical co-registration of the two modalities to record the variance in each pixel. Equations that use the transmission intensity to normalize SRS signals were developed. The effect of the normalization was assessed, with a focus on investigating and discussing the effect of reducing the SRS signal variance. Tretinoin was used as a model API for testing the capabilities of the developed method, and the method’s ability to aid in a BE assessment was also evaluated.

## 2. Materials and Methods

### 2.1. Materials

Retinoic acid (product number: R2625), polyethylene glycol (PEG-400), ethanol, and dichloromethane (DCM) were purchased from Sigma-Aldrich (Saint Louis, MO, USA). Poly(n-propyl methacrylate) (PPMA, approximate molecular weight = 150,000) was purchased from Scientific Polymer Products (Ontario, NY, USA). Retin-A^®^ 0.1% cream (NDC No. 0187-5164-20, Bausch Health US, LLC., Bridgewater, NJ, USA) and tretinoin 0.1% cream (NDC No. 0574-2201-20, Padagis US, LLC., Minneapolis, MN, USA) were purchased from WEP clinical (Morrisville, NC, USA).

The abdominal skin used in this research was obtained from the Cooperative Human Tissue Network (CHTN). The use of de-identified human skin tissue was approved by the Massachusetts General Hospital Institutional Review Board (IRB) as being “exempt, non-human” research. Abdominal skin from four donors (one 33-year-old female, one 47-year-old female, one 55-year-old female, and one 55-year-old male) was used in this study. A similar procedure to that employed in previous studies was used to section the skin into 1.5 cm × 1 cm pieces and store them in a freezer [26].

### 2.2. Instrumentation

This work used an SRS microscope comprising an inverted microscope (Olympus FV3000, from Olympus, Tokyo, Japan), an all-fiber OPO laser (Refined Laser Systems, Münster, Germany), an SRS/transmission detector built in-house, and mirrors and filters mounted in free space. The laser had a short wavenumber switching time (<5 ms) and a wide tunable range covering 700–3300 cm^−1^ wavenumbers. The imaging system enabled an automated process for moving to multiple regions of interest (ROIs) and depths, as well as switching to specified Raman shift wavenumbers [23]. Two SRS systems with two individual all-fiber OPO lasers were used. The same optical pathway and microscope were used in these two SRS systems. The main differences between the data measured using the two SRS systems were the laser powers and the lock-in-amplifier settings. The laser power varies across different wavenumbers. At the 1586 cm^−1^ used in this work, the pump and Stokes lasers’ powers in the sample were around 48.3 mW and 33.3 mW, respectively, when using laser No. 1; the two lasers’ powers were around 48.0 mW and 51.6 mW, respectively, when using laser No. 2. All the data were collected using a 20× objective with a 0.8 NA. A detector was positioned on top of the microscope condenser (0.55 NA) to detect the Stokes beam transmitted through the samples.

The SRS/transmission detector built in-house [39] was composed of a 1 cm × 1 cm silicon PIN photodiode and a circuit to separate the average- (DC) and high-frequency (AC) photocurrents, as well as respective transimpedance amplifiers for the DC and AC outputs, allowing for simultaneous measurement of the transmission and SRS signals. Ferrite core rings were used to suppress parasitic signals in the DC output wire and the power supply wire, preventing noise from reaching the sensitive transimpedance amplifiers. The DC signal was directly connected to an analog-to-digital converter and then read into the Olympus software. The AC signal was connected to a lock-in-amplifier (Moku:Lab, from Liquid Instruments, San Diego, CA, USA). The following settings were used in the lock-in-amplifier: the input impedance was 50 Ω; the time constant was 1.59 µs; a first-order low-pass filter (6 dB per octave) was used; the “sync” output from the laser source was used as the reference input; and the offset and the gain were adjusted according to the samples to achieve a high-contrast image without saturation. The demodulated SRS signal was amplified using a gain block, connected to an analog-to-digital converter, and then read into the Olympus software (version number 2.6.1.243). All images were collected using a scan size of 1024 × 1024 pixels and a scanning speed of 10 µs/pixel without line or frame averaging.

### 2.3. SRS Spectra Measurement and Photostability Test

The tretinoin formulations used in this study were Retin-A^®^ 0.1% cream (NDC No. 0187-5164-20, Bausch Health US, LLC, Bridgewater, NJ, USA), tretinoin 0.1% cream (NDC No. 0574-2201-20, Padagis US, LLC, Minneapolis, MN, USA), and a lab-made formulation (0.1% *w*/*w* tretinoin, 15.3% *w*/*w* ethanol, 84.6% *w*/*w* PEG-400). The SRS spectra of each formulation and of each excipient of each formulation were collected. These samples (i.e., creams and solutions) were sandwiched between glass cover slips and imaged at the sample and air interface from 1100 cm^−1^ to 1700 cm^−1^ with a step size of 4 cm^−1^. The background-adjusted SRS spectra were plotted using the SRS signal obtained from the sample divided by the signal obtained from a region in the same field of view with the sample absent.

The experiments conducted to confirm the photostability of the tretinoin formulations are described in the Supplementary Information.

### 2.4. Skin Preparation

A procedure similar to that used in previous studies was used to trim and prepare the skin samples for each experiment [26]. Briefly, a 1.5 cm × 1 cm frozen skin sample was thawed at room temperature and rinsed with phosphate-buffered saline (PBS). The skin was then placed on a plastic Petri dish with the stratum corneum side facing down. Forceps and microsurgical scissors were used to trim the skin to be thin and translucent enough for light transmission. The trimmed skin piece was cut into three sections with a 0.5 cm × 1 cm size.

### 2.5. Reference Polymer Film Preparation

Tretinoin-embedded PPMA polymer films were made as previously described [26]. In brief, to make a polymer film with 1% *w*/*w* tretinoin, 0.5 mg of tretinoin and 50 mg of PPMA were dissolved in 1 mL of DCM. A total of 80 µL of this mixture was then dried into a circular disk with an 8 mm diameter, which was stored in a small tube with a cap until use.

### 2.6. Skin Thickness Study

To assess how the transmission and forward-direction SRS signals changed due to variance in the skin thickness, one skin sample (55-year-old female) was purposefully trimmed to have non-consistent thicknesses. Four different spots on the skin were measured using a caliper, which showed that the thickness was in the range of 0.329–0.535 mm with an average value of 0.416 mm. Different concentrations of tretinoin reference polymer films were positioned under the skin. At least 16 ROIs on each reference polymer film were imaged. The dual-modality detector was used to collect transmission and SRS images simultaneously.

### 2.7. Formulation Thickness Study

Changes in the transmission and forward-direction SRS signals due to the different optical features and different thicknesses of the formulation were studied. Retin-A^®^ 0.1% cream and tretinoin 0.1% cream (Padagis US, LLC., Minneapolis, MN, USA) were used as samples with low transparency and high scattering. The lab-made solution with 0.1% *w*/*w* tretinoin in ethanol and PEG-400 was used as a sample with high transparency and low scattering. The formulation was smeared between two cover slips. Formulations with different thicknesses were made by adding microscope slide spacers with different heights between the two cover slips. The accurate thickness of the formulation was determined by using a caliper to measure the thickness of the two cover slips before and after adding the spacer. The dual-modality detector was used to collect transmission and SRS images simultaneously. The following thicknesses of the Retin-A^®^ 0.1% cream were measured: 10 µm, 53 µm, 90 µm, 107 µm, and 130 µm. The following thicknesses of the tretinoin 0.1% cream were measured: 17 µm, 60 µm, 83 µm, 113 µm, and 133 µm. The following thicknesses of the lab-made solution were measured: 90 µm, 97 µm, and 130 µm. In the experiment measuring different thicknesses of Retin-A^®^ on skin, 10 µm and 80 µm of the Retin-A^®^ 0.1% cream were used.

### 2.8. Bioequivalence Study

Retin-A^®^ 0.1% cream is a reference listed drug (RLD). Two groups of experiments using this formulation were performed and labeled as R1 and R2, respectively. A comparison of groups R1 and R2 was used to evaluate the inter-experimental variability in the same formulation. Tretinoin 0.1% cream (Padagis US, LLC., Minneapolis, MN, USA) is an approved generic topical product, which has been demonstrated to be BE to the RLD. The group of experiments investigating this generic product was labeled as group T. The lab-made formulation with 0.1% *w*/*w* tretinoin in ethanol and PEG-400 was designed to have distinct excipients compared to the RLD. The use of distinct excipients was expected to result in different BA from that of the RLD. This group of experiments using the lab-made formulation was labeled as group Lab. In total, four groups of experiments were performed: R1, R2, T, and Lab.

Every group of experiments was performed on ≥4 independent abdominal skin samples from each of the 4 skin donors. In one experiment, two randomly selected formulations were tested as a pair. A total of 5 µL of each formulation was applied on each 0.5 cm × 1 cm piece of skin. A standard reference polymer film with 1 mg tretinoin per 100 mg poly(n-propyl methacrylate) (PPMA) was put on a third piece of skin. All three skin samples were positioned in a glass-bottom well plate with the stratum corneum side facing down.

These three pieces of skin were imaged in sequence in one imaging cycle. The imaging cycle included three ROIs on the skin section with formulation 1 applied to it, three ROIs on the skin section with formulation 2 applied to it, and one ROI on the skin section with the standard reference polymer film applied to it. At each ROI, the sample was imaged in a Z-stack from a depth of 0 µm to 80 µm, with a step size of 8 µm. In the skin with the formulations applied to it, the 0 µm depth point was the uppermost layer and was defined as the point where the lipid-resonant signal of the hexagonal corneocyte structure was present. In the skin with a standard reference polymer film applied to it, the 0 µm depth point was determined to be the focus position at which the brightest C-H stretching signal (at 2870 cm^−1^) of the polymer occurred.

Permeation monitoring was carried out by repeating five imaging cycles tuned to 1586 cm^−1^, followed by one imaging cycle tuned to 2870 cm^−1^, until a total of 24 imaging cycles was reached. Each imaging cycle took 17 min to measure the Z-stacks of 7 ROIs (at depths from 0 to 80 µm). The images collected at 1586 cm^−1^ were used to measure the tretinoin within the skin. A major contribution to the signal at this peak was attributed to C=C stretching in tretinoin. The images collected at 2870 cm^−1^, attributed to C-H stretching, were used to measure the lipids within the skin to show the skin’s microstructure. Using the dual-modality detector developed in-house, the SRS and transmission signals in each pixel were collected simultaneously and recorded in an image with two channels. The 24 imaging cycles translated to an experimental length of approximately 6.8 h. During the study period, the well plate was kept in a microscope stage-top incubator (Tokai Hit INUG2F-IX3D, TOKAI HIT, Fujinomiya-shi, Shizuoka-ken, Japan) with a constant high humidity level (90%) and temperature (32 °C).

### 2.9. Image Processing and Statistical Analysis

In the ‘Skin Thickness Study’ and ‘Formulation Thickness Study’, the raw SRS value (SRS_Raw_) was obtained after averaging all the non-zero-value pixels in the SRS image, and the raw transmission value (Transmission_Raw_) was obtained after averaging all the non-zero-value pixels in the transmission image. The transmission-normalized SRS value was obtained using the correction equation SRS_TransNorm_ = SRS_Raw_ − a × (Transmission_Raw_ − b). The same correction equation with different optimized ‘a’ and ‘b’ values was used in this paper. The development of the equation and optimization of the ‘a’ and ‘b’ values are described in Section 3.3.

In the BE study, the collected time series of image stacks were processed to obtain “intensity–time profiles”. The “raw SRS intensity–time profile of the whole region” was obtained by calculating the raw SRS intensity at each time point after averaging the pixels of each 1586 cm^−1^ SRS image. Similarly, the “raw transmission intensity–time profile” of the whole region was obtained from the transmission images.

Two methods were used to normalize the SRS images using the transmission value. The first involved normalization using the transmission value of each ROI: the raw SRS value in each pixel and the transmission value from the first time point in the “raw transmission intensity–time profile” were used in the correction equation SRS_TransNorm_ = SRS_Raw_ − a × (Transmission_Raw_ − b). In this “normalize by ROI” method, the pixels in an SRS image were normalized using a single transmission value. The second method was normalization using the transmission value of each pixel: the raw SRS value in each pixel and the transmission value of the corresponding pixel were used in the correction equation. This pixel-by-pixel normalization was called “normalize by pixel”. All the non-zero-value pixels in the transmission-normalized SRS image were averaged to obtain the intensity–time profile. Because of the linear relationship to Transmission_Raw_ in the correction equation, the values after averaging the pixels in normalized SRS images using “normalize by ROI” and “normalize by pixel” are mathematically the same. Therefore, only normalized-by-pixel SRS images were averaged to obtain the intensity–time profile, which was denoted as the “pixel-wise transmission-normalized SRS intensity–time profile of the whole region”. A deep learning model-based method, which has been discussed in previous studies [11,12,26], was used to obtain “intensity–time profiles” for the lipid-rich intralamellar region and the lipid-poor corneocyte intracellular region. First, the 2870 cm^−1^ SRS image at a specific Z-depth and of a specific ROI on a given piece of skin was segmented into the lipid-rich intralamellar region and the lipid-poor corneocyte intracellular region. The segmented image dividing the lipid-rich and lipid-poor regions was then masked onto the 1586 cm^−1^ images from the previous 5 imaging cycles, located at the same Z-depth and ROI. All the non-zero-value pixels of the masked 1586 cm^−1^ SRS images were averaged to generate “raw SRS intensity–time profiles” in the lipid-rich intralamellar region and the lipid-poor corneocyte intracellular region. To obtain “pixel-wise transmission-normalized SRS intensity–time profiles” in the lipid-rich intralamellar region and the lipid-poor corneocyte intracellular region, the normalized-by-pixel SRS images were separated into two regions via the mask generated by the 2870 cm^−1^ SRS image. All the non-zero-value pixels of the normalized-by-pixel SRS images in each region were averaged to obtain the “pixel-wise transmission-normalized SRS intensity–time profile in the lipid-rich intralamellar region” and “pixel-wise transmission-normalized SRS intensity–time profile in the lipid-poor corneocyte intracellular region”.

The image stacks collected from the ROI with the standard reference polymer film were processed to obtain the “standard trace”. The SRS intensity value at a specific time point was calculated by averaging the SRS images taken from 0 µm (polymer film surface) to 24 µm (deep in the polymer) and then averaging all the pixels of the averaged image. The same calculation was repeated for images at all the time points to obtain the “SRS signal standard trace”. Similarly, the “transmission signal standard trace” was calculated using the transmission images.

Development of the correction equation and optimization of the ‘a’ and ‘b’ values are described in Section 3.3. In the BE study, specifically, the following ‘a’ and ‘b’ values were used in the correction equation: the ‘a’ value was 4.2 for bare skin and skin with a transparent lab-made formulation applied to it; the ‘a’ value was 1.6 for skin with tretinoin cream products applied to it (tretinoin 0.1% cream from Padagis US, LLC., and Retin-A^®^ 0.1% cream). In one experiment, the transmission normalization for ROIs with topical formulations applied to them used the transmission signal from the standard reference polymer film ROI in the same experiment. Specifically, a ‘b’ value equaling the value at the first time point in the “transmission signal standard trace” was used in transmission normalization for the ROIs in this experiment.

The previously obtained “raw SRS intensity–time profile” was divided by the “SRS signal standard trace” to obtain the “standard film-normalized SRS intensity–time profile”. The “pixel-wise transmission-normalized SRS intensity–time profile” was divided by the “SRS signal standard trace” to obtain the “standard film- and pixel-wise transmission-normalized SRS intensity–time profile”, which will be referred to as the “combined normalized SRS intensity–time profile” in the following sections. The steps taken to obtain these profiles are summarized in Figure S6 in the Supplementary Information. These “combined normalized” intensity–time profiles were used to calculate the area under the curve (AUC), maximum concentration (C_max_), and time at which maximum concentration occurs (t_max_) using non-compartmental analysis (NCA) in R [40]. The AUC was calculated using a linear–log trapezoidal approach, which used linear-up and log-down in the interpolation of the intensity–time curve. The AUC_0–6h_ value was used in this study.

The BE analysis method was based on confidence intervals and was similar to that used in a previous study [26]. Briefly, the AUC and C_max_ were firstly log-transformed. A pair of formulations were then compared by inputting their log-transformed AUC or C_max_ values as mentioned in the FDA guidance for IVPT studies [41]. The confidence interval (CI) was calculated using the regular average BE (ABE) calculation method mentioned in the FDA guidance for IVPT studies [41]. The outputted 90% CI for the mean difference between the two formulations was compared with the BE limits of [0.8, 1.25]. If either side of the interval exceeded the BE limits, the two formulations were considered non-bioequivalent. If the CI was within the BE limits, the two formulations were considered bioequivalent.

## 3. Results and Discussion

### 3.1. SRS Spectra and Photostability of Tretinoin Formulations

In the BE study, three different formulations with the same concentration of tretinoin were tested: Retin-A^®^ 0.1% cream, tretinoin 0.1% cream, and a lab-made formulation (0.1% *w*/*w* tretinoin, 15.3% *w*/*w* ethanol, 84.6% *w*/*w* PEG-400). The workflow shown in Figure S1 was used to better apply SRS imaging in the BE study. First, the SRS spectrum of the API was collected. As shown in Figure S2, tretinoin has a strong SRS peak at around 1586 cm^−1^, which is attributed to C=C stretching.

When imaging skin upon which a topical formulation has been applied, the skin and the excipients in the topical formulation can generate an SRS signal that may interfere with the signal from the target API. Therefore, the SRS signals of these non-API components need to be evaluated to ensure that the tretinoin signal is more prominent than the non-tretinoin signal contributions. The quantitative composition of all the excipients except for the API is usually accessible only to those who study self-made formulations or to the manufacturers who make the products. In these cases, the SRS spectrum of a placebo sample with identical excipients to the topical formulations can be directly collected. However, when information about the quantitative composition of all the excipients is not available, an alternative method needs to be used. As shown in Figure S1, one approach is to collect the SRS spectra of all the excipients and, thereby, confirm the specificity of the API’s signal. Alternatively, the chemical structure of the identified API’s SRS peak can be used to pinpoint the excipients that are likely to interfere with the API’s signal. Using this alternative method, only the SRS spectra of a subset of the excipients present with a specific chemical structure need to be measured.

**Figure 1:**
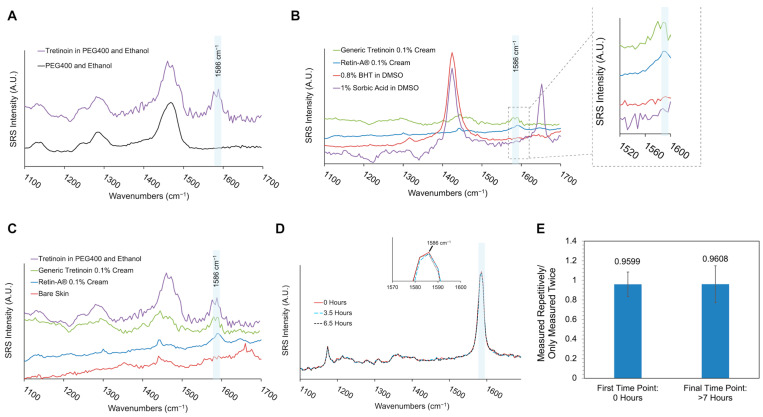
SRS spectra of formulations with tretinoin and the excipients in the formulations: (**A**) a 0.1% lab-made formulation containing PEG 400 and ethanol and (**B**) a Retin-A^®^ 0.1% cream and generic tretinoin 0.1% cream containing butylated hydroxytoluene (BHT). The spectra were shifted in the *y*-axis to avoid overlaps. (**C**) SRS spectra of the formulations and an ex vivo human skin sample. (**D**,**E**) Signal stability of tretinoin in SRS imaging: (**D**) SRS spectra of tretinoin powder under continuous SRS imaging for 0 h, 3.5 h, and 6.5 h. The inset is a zoomed-in view of the spectrum, showing the peak at around 1586 cm^−1^. (**E**) The SRS signal was measured from 0.1% tretinoin dissolved in DMSO at 1586 cm^−1^. Only measured twice: The sample was measured at the first time point and the final time point. Measured repetitively: The sample was measured repetitively 35 times from the first time point (0 h) to the final time point (>7 h). The error bar shows the standard deviation of four replicates.

In this study, the excipient-only (solvent) sample of the lab-made formulation was prepared, and its SRS spectrum was acquired (Figure 1A). For the two commercial topical products, there is no accessible cream base that has an identical quantitative composition to that of the commercial formulations, and hence, the chemical structures of all the listed ingredients were examined. As shown in Table S1, butylated hydroxytoluene (BHT) and sorbic acid were the two excipients that had C=C structures among all the ingredients. A BHT sample with a concentration of 0.8%, which is higher than the maximum concentration used in topical products according to the FDA’s inactive ingredient database, was used. In addition, 1% sorbic acid was used because this concentration is slightly higher than the reported concentrations used as a preservative in topical products. As shown in Figure 1A,B, no excipients had an appreciable signal near the 1586 cm^−1^ peak of the API. As shown in Figure 1C, the skin had a primary peak near 1660 cm^−1^, which was the peak attributed to the amide I bonds in human skin. In excised human skin, the 1586 cm^−1^ wavenumber band was located at the shoulder of the 1660 cm^−1^ peak, similarly to in the observation of in vitro skin in previous work [42]. The peak signal of the 0.1% tretinoin products was significantly more prominent than the skin’s endogenous contribution at 1586 cm^−1^. This allows for imaging of tretinoin applied to skin, with the acknowledgment that there may be a small endogenous contribution to the signal intensity from the skin background. These results confirmed the utility of the SRS signal of the 1586 cm^−1^ peak in imaging tretinoin versus non-tretinoin contributions (skin and excipients within the topical formulations).

In contrast with other APIs in topical products, tretinoin has poor photostability. Tretinoin undergoes photodegradation under sunlight and UV irradiation [43,44,45]. It has been reported that after 8 h of exposure to UV irradiation, more than 60% of the drug has undergone degradation [46]. Optical imaging techniques have not been extensively used in measuring tretinoin, which may partially be due to tretinoin’s photoinstability. To confirm the feasibility of using SRS imaging for continuous and repetitive imaging of tretinoin, multiple experiments were performed to assess the photostability of tretinoin formulations under repetitive SRS imaging. In one experiment, tretinoin powder was exposed to the laser for more than 6 h continuously. As shown in Figure 1D, no changes were observed in the peak position and intensity at around 1586 cm^−1^ in the three collected spectra, indicating the stability of the C=C bond of tretinoin during SRS imaging.

In the other experiment, two samples of 0.1% tretinoin dissolved in DMSO were used. One sample was used to collect 35 repetitive SRS images: the first image was taken at 0 h, and the final image was taken at a time point over 7 h later. The other sample was only exposed to the laser for collection of two SRS images: one image was collected at 0 h, and the other image was collected at a time point over 7 h later. This sample was not exposed to lasers between the two SRS image collections. The ratio of the SRS intensity collected using “repetitive measurements” to that collected using “only two measurements” at the first time point and the final time point were measured, calculated, and compared. As shown in Figure 1E, the difference in the results between the two methods was minimal. In addition, according to the Shapiro–Wilk normality test and Levene’s test, the two datasets were normally distributed and had equal variance; the results obtained using “repetitive measurements” and “only two measurements” were not statistically significantly different based on a *t*-test. This result showed that repetitive SRS imaging does not degrade the C=C bond of tretinoin.

The above experiments used the SRS signal, which primarily probed the stability of the C=C stretching of tretinoin. Moreover, LC-MS was used to confirm the stability of the tretinoin molecule after 7 h of continuous exposure to lasers during SRS imaging.

The LC-MS results confirmed that the tretinoin concentration in the creams after 7 h of repetitive SRS imaging was close to the tretinoin concentration in the creams without laser exposure: the ratio of concentrations of tretinoin detected under these two conditions was 1.03 ± 0.09 (*n* = 3) for the Retin-A^®^ 0.1% cream and 1.02 ± 0.06 (*n* = 3) for the tretinoin 0.1% cream.

The results from the above experiments confirmed the signal’s stability during repetitive SRS imaging, a finding which may have been due to the near-infrared wavelength of the lasers used in SRS imaging (>800 nm), which is far from the UV wavelengths that cause the reported photodegradation.

### 3.2. Dual-Modality Detector for SRS and Transmission Signals

**Figure 2:**
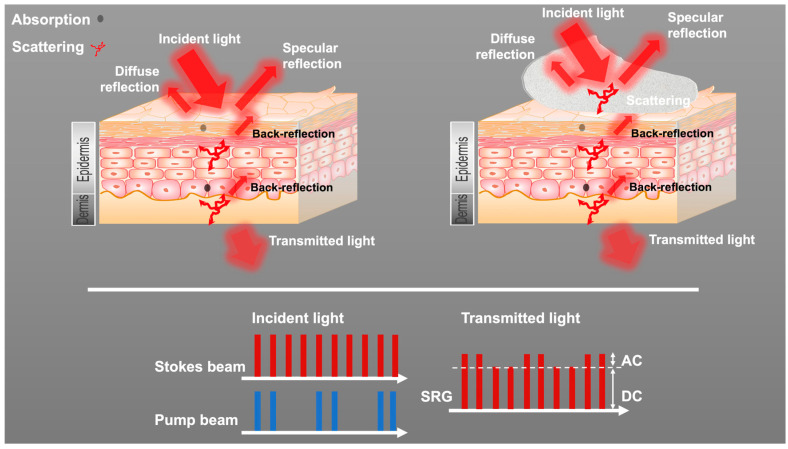
Schematic representation of optical processes occurring during light propagation in skin samples. **Left**: Light propagates through a skin sample. **Right**: Light propagates through a skin sample with a cream formulation applied to it. Incident light includes a Stokes beam and a modulated pump beam. The transmitted Stokes beam is measured and the SRG is measured using a lock-in-amplifier.

As shown in Figure 2, multiple processes occur between the incident light entering the skin and the light being measured on the other side of the skin. The light intensity on the other side of the skin is related to the incident light’s intensity and is dependent on the skin’s thickness and pigmentation variance. In addition, the optical features of the applied formulations also alter the measured transmitted light intensity. Because the macroscale control of the skin thickness, skin tones, and formulation thickness is coarse relative to the microscale size of the imaging ROI, control of the experimental conditions is limited for reducing the variance contained in the transmitted SRS signal. Therefore, a novel method capable of pixel-wise normalization was developed to solve this problem.

In this work, the SRS/transmission detector built in-house output the AC and DC signals simultaneously. The AC output was demodulated to obtain the stimulated Raman gain (SRG) signal, which was then amplified, converted, and used to generate the SRS image. The DC output was directly connected to an analog-to-digital converter and used to generate the transmission image. The AC and DC signals originated from the same incident laser, were generated at the same spot on the sample, traversed the same optical path, and were collected by the same photodiode. These features ensured that the measured AC and DC outputs were both synchronized in time and co-registered in space, making the SRS image and the transmission image match pixel by pixel. Moreover, because the SRS and transmission signals were measured from the same light beam, they underwent the same scattering and absorption processes when propagating in the skin samples.

It is worth mentioning that the generation of an SRS signal is a nonlinear process and the SRS intensity is the product of the pump beam and the Stokes beam at the focal point. If scattering and absorbance-caused light variance occur before the focus, both the pump beam and Stokes beam may be perturbed and the effect can be nonlinear. The imaging in this work was carried out at a relatively shallow depth (0–16 µm) in the skin, so this work did not attempt to address this potential loss. Instead, a methodology was developed to compensate for the losses of the transmitted beams. In the transmitted optical path, the impact of light scattering is a linear process, where photons are scattered out of the transmission beam path. As the SRS process leads to a transfer of the intensity via Raman gains/Raman losses, the SRS signal and transmitted light signal experience the same losses and therefore have a linear relationship. In this work, the stimulated Raman gain was measured using the Stokes beam, so the Stokes beam alone contained sufficient information to compensate for the measured SRS signal’s variance.

**Figure 3:**
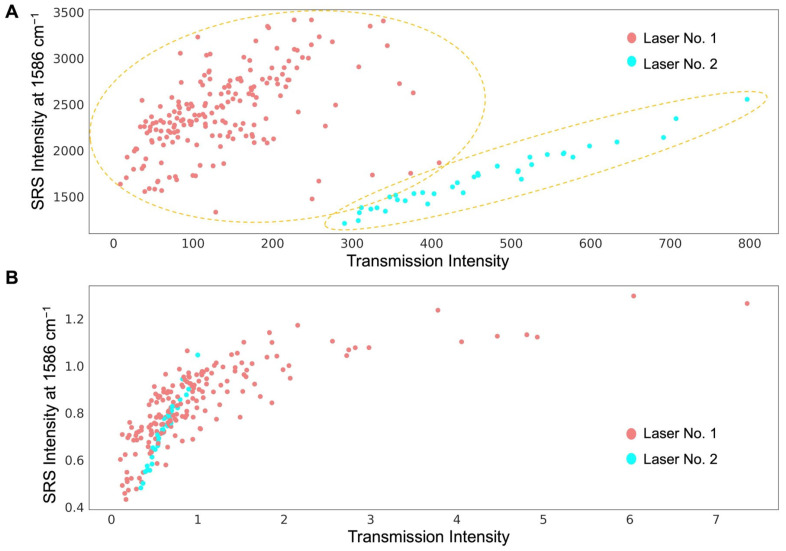
(**A**) SRS and transmission intensities at the first time point after applying a topical formulation on an ex vivo human skin sample. Each dot represents an ROI in an experiment applying a formulation on a skin sample from a donor. The points include four groups of experiments using different formulations: R1, R2, T, and NC. Each group was tested on ≥4 independent abdominal skin samples from each of the 4 skin donors (33-year-old female, 47-year-old female, 55-year-old female, and 55-year-old male). Laser No. 1 refers to the data collected from one SRS imaging system, and laser No. 2 refers to the data collected from the other SRS imaging system. The SRS signal at 1586 cm^−1^ was used. (**B**) SRS and transmission signal intensity after normalization to the signal from the reference polymer film.

In a previous study [26], a standard reference polymer film was developed and used for SRS signal normalization. The formulation ROI’s SRS signal was normalized to the polymer film’s SRS signal using SRS_Raw_/SRS_StandardReferenceFilm_. In this study, a polymer film embedded with tretinoin was also used to normalize the SRS signal, which is referred to as “standard film normalization”. Tretinoin experiments were performed on two different SRS imaging systems with the same optical design but different laser sources. The laser powers and the lock-in-amplifier settings were different between the two lasers. As shown in Figure 3A, the intensities of the two systems are clearly distributed in two separate clusters. After normalizing the sample signal to the polymer film’s signal, the difference in the SRS and transmission intensities between the two systems was greatly reduced (Figure 3B). The standard film normalization method is helpful for reducing signal variance caused by changes occurring in the optical setup/laser power between experiment days or by differences in the skin pigment content and/or skin thickness between donors, samples, and experiments.

However, because the standard film ROI was separate from formulation ROIs, the microscale heterogeneity between the ROIs and/or pixels cannot be well considered or compensated for using the standard film normalization method. The high-transmission and high-SRS points corresponding to data obtained using laser No. 1 in Figure 3B may be related to some experiments where there was a large difference in the scattering/absorbance between the formulation ROIs and standard film ROIs. For example, the skin region in the standard film ROI could have had a larger thickness or contained more melanin than that in the formulation ROI.

### 3.3. Normalization of SRS Signal Using Transmission Signal

As mentioned above, standard film normalization has a limited ability to normalize signals when there is a large difference in the scattering/absorbance between the formulation ROI and the standard film ROI. The skin’s microscale heterogeneity and the optical features of the applied formulations are major factors contributing to this difference. The exact contribution of these factors cannot be determined in each measurement. However, while the light transmission was acquired together with the SRS signal, it can be used to compensate for the variance caused by these factors. Experiments varying the skin’s microscale heterogeneity and the optical features of the applied formulations were carried out to investigate the relationship between the SRS intensity and the transmission intensity. The observed data was used to develop an empirical equation for normalizing the SRS signal using the transmission signal, with the goal of minimizing the observed experimental variance.

**Figure 4:**
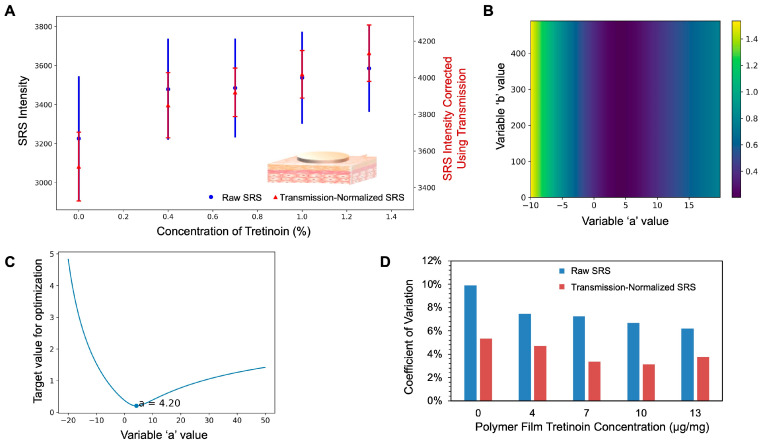
(**A**) SRS intensities of reference polymer films with different concentrations positioned under the skin. Skin thickness was measured using a caliper at four different spots. The thickness values were within 0.329–0.535 mm, with an average value of 0.416 mm. The primary *y*-axis shows the raw SRS intensity. The secondary *y*-axis shows the transmission-normalized SRS intensity. The raw SRS signal was measured at 1586 cm^−1^. Transmission-normalized SRS intensity was calculated using the empirical correction equation with a = 4.2 and b = 348. (**B**) Optimization of the parameters ‘a’ and ‘b’ in the empirical correction equation to minimize the target value, which was the sum of the CV values of the SRS intensities calculated for each concentration. The color bar represents the target values corresponding to various colors. (**C**) Optimization of parameter ‘a’ in the correction equation to minimize the target value, when specifying that b = 348. The value 348 is the transmission intensity of one data point measured from 0% tretinoin polymer film. (**D**) CV values calculated using the SRS intensities from polymer films containing different concentrations of tretinoin. Transmission-normalized SRS intensity was calculated using the empirical correction equation with a = 4.2 and b = 348.

First, a simpler case using bare skin without the application of any formulations was studied, where polymer films with different tretinoin concentrations were employed. Polymer films were used because the concentration of the embedded tretinoin could easily be controlled and they provided a stable, constant, and optically clear standard sample. The relationship between the SRS intensity and the concentration for these polymer films is shown in Figure S3, displaying a linear increase in the SRS signal with the tretinoin concentration. The polymer films were positioned under bare, untreated skin samples with purposefully high degrees of heterogeneity in their thickness. In contrast to artificial skin models, it is challenging to control the presence of various microscale structures in ex vivo human skin. Instead, the thickness of the trimmed skin samples, which was related to the extent to which the dermis layers were removed, was varied to introduce heterogeneity. The thinner region of the skin was expected to largely contain viable epidermis and less of the dermis. On the contrary, the thicker region of the skin was expected to contain more dermis, which would mean the presence of more skin structures like papillary dermis and sebaceous glands and more media to scatter and absorb light. Because of the introduction of microscale heterogeneity in the skin, large SRS variance was observed in the measured raw SRS signals (Figure 4A, blue points). The variance in the raw SRS signal was larger than the SRS intensity differences that would arise due to small differences in the tretinoin concentrations.

Both the transmission intensity and transmitted (forward-direction) SRS intensity are susceptible to variance in the skin thickness. As shown in Figure S4A, a linear correlation between the SRS intensity and transmission intensity was observed: thicker skin led to lower transmission and also lower SRS. In this experiment, the film was positioned and measured on the surface of the skin, where the scattering and absorbance mainly perturbed the generated SRS signal. When the generated SRS signal passes through different thicknesses of skin after the microscope focus, the intensity loss can be treated as a linear process, which matches the observed relationship between the SRS intensity and transmission. Considering the linear relation between the SRS and transmission intensities measured from the tretinoin polymer film, two skin measurements have the phenomenological relation below:

(1) $$\frac{\left({SRS-SRS}^{\prime }\right)}{\left(Transmission-{Transmission}^{\prime }\right)}=a$$

which can be rearranged to

(2) $${SRS}^{\prime }=SRS-a\times \left(Transmission-{Transmission}^{\prime }\right)$$

In the above equation, ‘a’ is a constant factor that scales the transmission changes with respect to the SRS changes. If a reference transmission value is specified as a constant, ‘b’, all the points in Figure S4A can be transformed to a new point using an empirical correction equation: SRS_TransNorm_ = SRS_Raw_ − a × (Transmission_Raw_ − b). As shown in Figure S4B, using the same reference transmission value, the data points with the lowest transmission and the highest transmission are brought closer together following transmission normalization.

A practical minimization search approach was used to determine the optimal ‘a’ and ‘b’ values. For data points measured from a tretinoin polymer film, an optimal correction equation should impart the highest reduction in the SRS intensity variance across measurements; doing so would mean that the equation has minimized the SRS intensity perturbations caused by skin thickness differences and the optical effects of the formulations. Here, coefficient of variance (CV) values were used to quantify the variance. A parameter search was conducted across data points acquired from polymer films with different concentrations of tretinoin (0–1.3% tretinoin *w*/*w*); the ‘a’ and ‘b’ values were systemically adjusted to minimize the sum of the CV values across the range of tretinoin film concentrations. Because the SRS intensity in Figure 4A is raw and has not been baselined, it contains background signals from electronic devices and SRS imaging (e.g., cross-phase modulation, two-photon absorption, and thermal effects) [47], which causes the non-zero value in 0% tretinoin.

As shown in Figure 4B,C, different ‘b’ values did not materially affect the sum of the CV value, while the optimal ‘a’ value was found to be important, with an optimal value of 4.2. Compared to the raw SRS intensity, the transmission-normalized SRS intensity obtained using the empirical correction equation showed much lower CV values (Figure 4D). The smaller intensity variance helped to differentiate between signals at different tretinoin concentrations, as shown in the comparison in Figure 4A. Therefore, the developed correction equation with suitable ‘a’ and ‘b’ factors helped to reduce the total variance along the SRS intensity axis to compensate for the effects of differences in the skin thickness.

**Figure 5:**
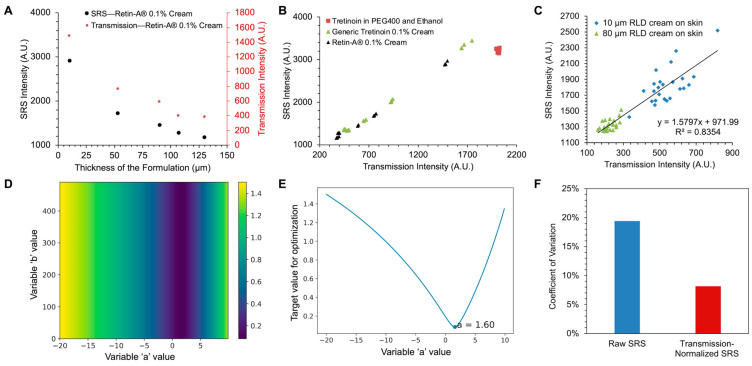
(**A**) SRS and transmission intensities of different thicknesses of Retin-A^®^ 0.1% cream. Signals were collected when focusing on the first layer of the cream, close to the objective. (**B**) Correlation between the SRS and transmission intensities of the Retin-A^®^ 0.1% cream, generic tretinoin 0.1% cream, and 0.1% lab-made formulation. Different thicknesses of the Retin-A^®^ 0.1% cream, generic tretinoin 0.1% cream, and lab-made formulations were imaged to collect the data points in this plot. (**C**) Correlation between the SRS and transmission intensities in skin with different thicknesses of Retin-A^®^ 0.1% cream applied to it. (**D**) Optimization of the parameters ‘a’ and ‘b’ in the empirical correction equation to minimize the target value, which was the sum of the CV values of the SRS intensities for the two tested thicknesses. The color represents the target value. (**E**) Optimization of parameter ‘a’ in the correction equation to minimize the target value, when specifying that b = 348. (**F**) CV values calculated using the SRS intensities for different thicknesses of Retin-A^®^ 0.1% cream. Transmission-normalized SRS intensity was calculated using the empirical correction equation with a = 1.6 and b = 348.

Topical formulations have different optical features, which may be caused by emulsion droplets, differences in the size of emulsion droplets, absorption effects, and thicknesses applied to the skin. If the formulation causes alterations to the SRS and transmission signal intensities, the empirical equation parameters must be adjusted. Among the three tretinoin-containing formulations, the RLD and generic products were both white and have similar-sized emulsion droplets in the cream. In terms of the thickness, it was found that various thicknesses of the RLD cream demonstrated different SRS and transmission intensities (Figure 5A). The thicker the sample, the lower the SRS and transmission intensities were. As shown in Figure 5B, the RLD and generic products showed the same linear correlation between the SRS and transmission intensities with varying cream thicknesses. The lab-made formulation, which was a transparent solution, did not show signal variance with varying solution thicknesses. For this reason, the empirical equation and parameters determined for untreated bare skin can be used for transmission normalization of the SRS signal from skin treated with the lab-made formulation.

In contrast, the equation determined for bare skin was not suitable for use with cream-treated skin due to the formulation’s light-scattering effects. Further experiments were carried out to investigate the relationship between the SRS and transmission intensities when the skin was treated with creams of varying thicknesses. As shown in Figure 5C, the application of a thicker formulation on the skin led to lower SRS and transmission and vice versa, as expected. These scattering-based effects demonstrated the same linear fit between the transmission and SRS intensities as that observed in untreated skin, such that the same linear equation as above could be used. As mentioned above, the generation of an SRS signal is a nonlinear process. If the scattering- and absorbance-caused light loss occurred before the focal point, both the pump beam and Stokes beam would be perturbed, and the transmission and SRS signal would have a nonlinear relationship. If the light loss occurred after the focal point, the transmission and SRS signal would have a linear relationship. In this experiment, the SRS and transmission signals were measured at the surface of the formulations with different thicknesses. In this case, transmission of the generated SRS signal was perturbed, and thus, a linear relation was observed in the data obtained. That is to say, the developed model is more precise for studying SRS signals generated on the surface of the sample. When the focal point is deep in the topical formulation, nonlinear processes would have a significant effect on the final detected SRS signal. A precise description of this process will require a more complicated nonlinear model and requires additional measurements other than the SRS and DC intensities. Considering this, the linear equation model was only applied in normalizing the SRS signal collected on the surface of the skin (0 µm) or in the top layers of the skin (0–16 µm) in this BE study.

The parameters ‘a’ and ‘b’ were determined by minimizing the sum of the CV of the SRS intensities for the two tested cream thicknesses. Figure 5D,E show that different ‘b’ values did not affect the sum of the CV values, while the optimal ‘a’ value was determined to be 1.6. Based on these results, the empirical correction equation can be applied with a = 1.6 to normalize the SRS signal measured from skin with the Retin-A^®^ 0.1% cream or the generic tretinoin 0.1% cream applied to it. As shown in Figure 5F, the transmission-normalized SRS showed significantly lower CV values compared to that observed for the raw SRS intensity. It is worth mentioning that a number of strategies to improve SRS imaging have been discussed in previous studies [48,49], mainly focused on reducing laser noise and improving the signal-to-noise ratio (SNR) in individual SRS images. The heterogeneity of transmission and scattering in samples has previously been taken into consideration when designing SRS detection schemes [48]. In one such scheme, when the Stokes beam was measured by a detector to extract the SRS signal, a reference beam with perpendicular polarization to that of the Stokes was measured by a second detector. This method was applied to thin tissue samples (30 µm), with the reference beam-corrected SRS images found to have an improved SNR. Such an approach, however, can only work in thin samples; the sample thickness used in this study would have led to polarization scrambling due to multiple scattering. Instead, the transmission modality was used in our work to empirically correct the absorption and scattering heterogeneity, especially that caused by different skin thicknesses (>300 µm) and the optical features of the applied topical formulations. Moreover, data from the additional output channel for transmission was recorded, which was not achieved in previous studies. Keeping this additional information enabled more versatile signal processing so that we could consider the impact of absorption/scattering heterogeneity. To the best of our knowledge, this is the first time that such an approach has been used to correct the SRS signal intensity for thick, heterogeneous tissue samples. Importantly, the detection scheme uses only a single detector with transmission and SRS outputs, which were easily added to the SRS imaging system without the need to alter the existing optical setup.

### 3.4. Bioequivalence Study of Topical Tretinoin Products Using Dual-Modality Detection

As discussed above, normalization of the SRS signal using the transmission signal helped to reduce the signal variance when measuring skin samples with a formulation applied to them. This section describes an exploratory BE study of topical products based on SRS imaging. The developed empirical equation was applied in the BE study, which used tretinoin as a model API, to assess its performance in the study.

For each individual ROI in an experiment, the “raw SRS intensity–time profile” was collected. After transmission normalization, “pixel-wise transmission-normalized SRS intensity–time profiles” were computed. After normalizing the ROI’s signal to that of the standard film, the “standard film-normalized SRS intensity–time profile” and “combined normalized SRS intensity–time profile” were obtained. The averages of all the ROIs of the samples in each treatment group were plotted as averaged profiles for the four studied groups (Lab, T, R1, R2). The averaged profiles are shown in the Supplementary Information (Figure S5).

The SRS values at the first time point in these intensity–time profiles of all the individual ROIs from the different treatment groups were used to calculate the mean, SD, and CV values. The SD and CV values helped to evaluate the effect of applying the correction equation on the SRS signal intensity. As shown in Table 1, combined normalization helped to reduce the signal variance observed in the raw SRS signal. Specifically, standard film normalization reduced the variance by 0.73%, 2.72%, and 3.46% in each treatment group and reduced the variance by 1.8% in all the formulation groups. Moreover, a combination of standard film normalization and transmission normalization reduced the variance by 5.83%, 8.76%, and 9.46% in each treatment group and reduced the variance by 7.85% in all the formulation groups. The variance decreases were much larger when using the combination method compared to using standard film normalization alone. These results confirmed that normalization using transmission effectively reduced the signal variance that occurred in SRS imaging-assisted topical product studies.

**Figure 6:**
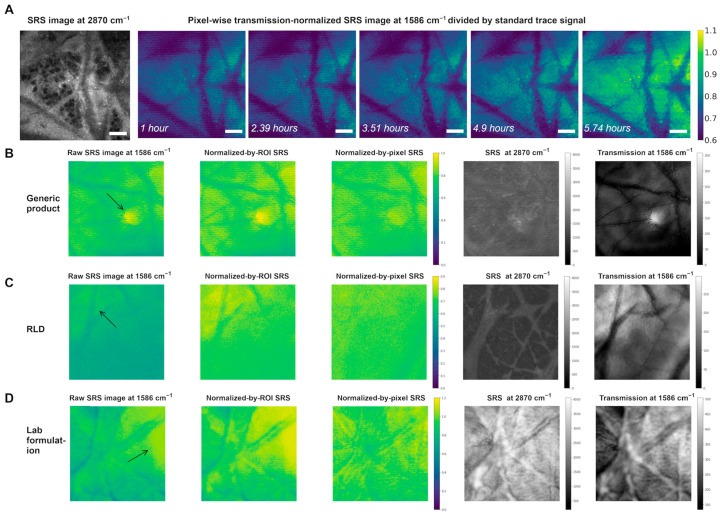
(**A**) SRS images showing the signal changes over time after applying the tretinoin RLD on the skin. The SRS images show the pixel-wise transmission-normalized SRS divided by the SRS signal standard trace from the standard polymer film ROI. The images were collected at a depth = 0 µm. (**B**–**D**) Images showing small areas of large heterogeneity. From left to right, the images are the raw SRS image divided by the standard trace signal, the normalized-by-ROI SRS image divided by the standard trace signal, the normalized-by-pixel SRS image divided by the standard trace signal, the raw SRS image showing the lipid microstructure, and the raw transmission image. The scale bar represents 100 µm. The same color bar is used for SRS images in the same row. The arrows drawn point to the regions where the SRS signal is heterogeneous and has strong signal intensity.

The drug distributions are visualized in Figure 6. As shown in Figure 6A, the SRS image at 2870 cm^−1^ highlights the lipid-rich intralamellar region and the lipid-poor corneocyte intracellular region. The drug permeation and accumulation on the skin was visualized using SRS images at 1586 cm^−1^ that were collected at different time points after applying the tretinoin formulation.

Some ROIs that had largely heterogeneous SRS signals are demonstrated in Figure 6B–D. In this work, an excitation objective with a 0.8 NA and a microscope condenser with a 0.55 NA were used. As the condenser had a lower NA than the excitation objective, light transmitted through the skin that was distorted or deflected due to scattering or by topical formulations may not have been captured by the detector. Appendages, as well as wrinkles and ridges, can cause distortion of the transmitted light and lead to areas with a reduced collected light intensity. The leftmost images in Figure 6B–D show a heterogeneous intensity and dark regions. Notably, the impact of these structures would impact both the SRS and transmission images, producing a feasible solution to reduce this heterogeneity by transmission normalization. In a sample with the generic product applied to it (Figure 6B), one ROI showed a small circular area with a much stronger SRS signal than those of the surrounding areas. A bright dot and very dark surrounding areas were also observed in the transmission image, which indicated that this ROI included a small, very thin area within a relatively thick area. The normalized-by-ROI SRS image was able to compensate for the higher average thickness of this ROI compared to that of the other ROIs or samples by increasing the intensity of the whole image. However, the heterogeneity between the small circular area and the surrounding areas remained. In contrast, the pixel-wise transmission normalization enabled a decrease in this heterogeneity, which resulted in a smaller SRS signal difference between the small circular area and the surrounding areas. Similarly, Figure 6C,D show more examples demonstrating the capability of pixel-wise transmission normalization to correct the SRS signal heterogeneity between a high-transmission area and the surrounding low-transmission areas. In general, compared to normalization by ROI, normalization by pixels had a better ability to capture some pixels’ drastic SRS and transmission changes.

We compared the results of the BE analysis based on SRS data performed using either standard film normalization alone, without transmission normalization, or a combination of standard film normalization and transmission normalization.

To carry out a BE analysis based on SRS data using standard film normalization alone, PK parameters (C_max_ and AUC_0–6h_) were extracted from the “standard film-normalized SRS intensity–time profile”, which were then used to calculate CIs (Table 2). The CIs of the C_max_ and AUC_0–6h_ were within the (0.8, 1.25) range when comparing R1 and R2, indicating good intra-group variance. When comparing R1 and the generic product or R2 and the generic product, the CIs were also within the BE range, which aligns with the expected BE between the RLD and the approved generic drug. When comparing R1 and Lab or R2 and Lab, the CIs exceeded the BE range, which indicates that the RLD and the lab-made formulation were not bioequivalent. This observation may have been due to the different excipients’ impacts on the API’s permeation. In addition, the different optical features of the white cream and clear lab-made solutions, as demonstrated above, may have contributed to the signal difference between these groups. The CI values of the lipid-rich intralamellar region were very close to those of the lipid-poor corneocyte intracellular region.

Another BE analysis was carried out based on SRS data using a combination of standard film normalization and transmission normalization. PK parameters (C_max_ and AUC_0–6h_) extracted from the “combined normalized SRS intensity–time profile” were used to calculate CIs. As shown in Table 3, the CI of the AUC_0–6h_ for the lipid-poor corneocyte region was (0.93, 1.07) when comparing R1 and R2. Compared to the CI calculated using standard film normalization alone (0.92, 1.17), the CI based on data obtained using the combination method was much narrower (a reduction of 0.11 in the CI range). When comparing R1 and R2, narrower CIs were observed for the C_max_ and AUC_0–6h_ in both the lipid-rich intralamellar region and the lipid-poor corneocyte intracellular region. These results indicate an improvement in the data quality achieved by reducing the intra-group variance. In the rest of the table, the BE between the RLD and the generic product is still clearly demonstrated by the CIs. The CIs when comparing R1 and Lab or R2 and Lab were slightly narrower than the values in Table 2, a result that was due to the reduction in the data variance caused by variability in the skin thickness and the different optical features of the applied formulations. After compensating for these factors, the CIs were still outside of the BE range. This result further confirmed the non-bioequivalence between the RLD and the lab-made formulation.

## 4. Conclusions

The SRS intensity measured in the forward direction varies due to the heterogeneity of skin in transmitting light and due to the various optical features of topical drugs applied on the skin. In this work, the effect of these factors on the SRS signal was studied in controlled experiments using skin and formulations with pre-determined thicknesses. It was observed that the SRS intensity decreased when the ex vivo skin samples were thicker. For transparent lab-made solutions, the SRS intensity did not change with the thickness of the solution. In contrast, the thickness of non-transparent topical creams had a conclusive impact on the SRS intensity. To reduce the data variance caused by these variables, an SRS/transmission dual-modality detector was introduced for use in SRS imaging, and empirical correction equations were developed and optimized. The correction equations used the SRS and transmission intensities, which were synchronized in time and co-registered in space. Pixel-by-pixel normalization using the transmission was successfully achieved.

Data from the tretinoin study was used to evaluate the improvement in BE assessment using the dual-modality method. The standard film normalization method alone helped to reduce the CV in the SRS signal (the decrease ranged from 0.7% to 3.5%). A combination of normalization using signals from the standard film and the transmission reduced the CV in the SRS signal more than the former method (the decrease ranged from 5.83% to 9.46%). Moreover, in BE analysis, compared to the CIs of the C_max_ and AUC_0–6h_ calculated using standard film normalization alone, the CIs based on data obtained using the combination method were much narrower. Specifically, the narrower CI observed when comparing the R1 and R2 groups indicated improved performance in reducing the intra-group variance. The expected BE between the RLD and generic product was confirmed by the calculated narrow CI. The expected non-BE between the RLD and lab-made formulation was also demonstrated by the CI exceeding the BE range.

The optical setup and the detection geometry used determine the impact of scattering effects on the measured SRS intensity. In this work, the condenser had a lower NA (0.55) than the excitation objective (0.8). In this particular case, light deflected due to scattering in the skin or topical formulations may not have been captured by the detector and thus caused signal loss. This effect in the experimental setup could be the reason for the large improvement observed after introducing transmission normalization. However, for a system with a much higher NA within the detection arm, this scattering-caused signal variance may be reduced. Considering this, the prominent effect of the developed normalization method is mainly applicable to SRS systems using similar NA values to those in this work.

It is also worth mentioning that this exploratory work as an initial step in utilizing transmission in SRS image processing. Instead of using complicated models/equations for a comprehensive expression of the SRS versus the transmission, this work was mainly focused on using a simple linear equation derived using an empirical method. The model/equation mainly considers the scattering effects that occur after SRS generation at the focal point. Thus, it is not suitable for correcting signal loss that happens before SRS generation. The current method is limited to normalizing SRS signals generated on the surface or in the very upper layers of the sample. The observed linear relationship is applicable within the signal range measured in the skin samples in this work. However, for different types of samples or samples with more drastically variable signals, such as skin with many heterogeneously distributed hair follicles and glands or highly heterogeneous pigmentation, a linear description may be inappropriate. More complicated models/equations will be needed to develop methods that can be generalized to diverse samples.

In this study, because tretinoin has a strong primary Raman peak, providing a good SRS signal, it was used as a model API to illustrate the capabilities of our method. Because the current method is limited to the surface of the skin, this work only explores its distribution in the upper layer. This point needs to be considered when interpreting the BE results from this study. In the future, a method that is capable of correcting light losses that happen both before the focus (pump and Stokes light loss) and after the focus (Stokes light loss) is needed for studying topical products that are pharmacologically active in deeper skin.

In addition, the developed method has other limitations. Firstly, the parameters in the empirical equation were determined based on controlled experiments using the skin and topical drugs used in the target BE study. The parameters cannot be directly extrapolated to other experiments. New samples or SRS systems would require the determination of their own optimal parameters using this work’s workflow. Secondly, this study is limited to light-skin-tone ex vivo skin samples, which rules out the changes that could occur for samples with different skin tones. Dark skin has an abundance of melanin, which strongly absorbs light. The variance caused by samples from donors with different skin tones needs to be assessed. Thirdly, this study mainly focused on using the transmission to reduce the SRS variance between individual samples for a bioequivalence comparison. As the focus of this developed method was the accurate and low-variance comparison of different treatment groups, it may not be suitable for the direct assessment of an API’s absolute concentration. Therefore, the developed model should only be used in comparative studies like a BE study. Considering the tight focus of SRS imaging, recovering the true ’pure’ SRS intensity and eliminating any signal losses due to light-scattering or -absorbing entities in each pixel should be feasible. Fourthly, this study did not correct for SRS signal attenuation at different depths, which is important for comparing the bioavailability of an API at different depths. Future work is needed to consider these aspects in order to further improve SRS imaging and expand its application. A comparative study comparing this SRS-based method and other methods for evaluating the local BE using various BE and non-BE topical products would be significant future work advancing the application of the SRS-based method.

This work investigated factors that could affect the SRS signal when applying SRS imaging in a topical drug study, which have not been well considered in previous studies. The observations in this work lead to a better understanding of these variables and the extent to which they can influence SRS imaging and SRS signals. Considering their impact, methods for eliminating or reducing their effects are essential for better application of SRS imaging in cPK studies of topical drugs. This work overcame this challenge and provided a method to reduce the data variance caused by these factors and inconsistencies. The dual-modality detector enabled simultaneous measurement of the SRS and transmission intensities resulting from light paths traversing the same sample and experiencing the same absorption/scattering losses. Normalization of the SRS signal using the transmission intensity based on empirical correction equations provided a straightforward and easily accessible solution to address the data variance problem.

## Figures and Tables

**Table 1:** Comparison of the data variance in the raw SRS intensity and normalized SRS intensity. The mean value and CV value for the 0.1% lab-made formulation were calculated using the intensity values at the first time point of all the ROIs in all the experiments involving the application of the lab formulation to the ex vivo human skin samples. ∆CV is the difference after subtracting the CV values in the raw SRS signal row.

Formulation		Lab	T	R1 and R2	All Formulations
Donor		All Four Donors	All Four Donors	All Four Donors	All Four Donors
Raw SRS signal	Mean	2443.78	2323.46	2212.42	2295.71
	SD	461.68	513.38	483.93	495.28
	CV	18.89%	22.10%	21.87%	21.57%
Standard film normalization: Raw SRS signal/ standard film	Mean	0.88	0.79	0.80	0.82
	SD	0.14	0.15	0.17	0.16
	CV	15.43%	19.38%	21.14%	19.82%
	ΔCV	−3.46%	−2.72%	−0.73%	−1.75%
Combined normalization: Pixel-wise transmission-normalized SRS signal/ standard film	Mean	0.96	0.84	0.86	0.88
	SD	0.12	0.11	0.11	0.12
	CV	13.06%	13.34%	12.41%	13.72%
	ΔCV	−5.83%	−8.76%	−9.46%	−7.85%

**Table 2:** BE analysis of the differences in the log-transformed C_max_ and AUC_0–6h_ between topical products in the upper skin layers. The C_max_ and AUC_0–6h_ were extracted from normalized SRS intensity–time profiles. The C_max_ and AUC_0–6h_ of four ROIs and three depth slices (0–16 µm) from a sample were averaged into one value before BE analysis. The CI was calculated using the regular average BE (ABE) calculation method mentioned in the FDA guidance for IVPT studies. The output CIs were converted back to non-log-transformed values and written as (low limit, high limit).

Product 1	Product 2	90% Confidence Interval for the Mean Difference in the C_max_ Values (Product 2, Product 1)		90% Confidence Interval for the Mean Difference in the AUC_0–6h_ Values (Product 2, Product 1)	
		Lipid-Rich Intralamellar Region	Lipid-Poor Corneocyte Region	Lipid-Rich Intralamellar Region	Lipid-Poor Corneocyte Region
RLD (R1)	RLD (R2)	(0.91, 1.11)	(0.91, 1.12)	(0.92, 1.16)	(0.92, 1.17)
RLD (R1)	Generic	(0.92, 1.11)	(0.91, 1.11)	(0.91, 1.13)	(0.90, 1.12)
RLD (R1)	Lab (PEG/ETOH)	(1.12, 1.33)	(1.12, 1.35)	(1.11, 1.37)	(1.12, 1.38)
RLD (R2)	Generic	(0.93, 1.11)	(0.92, 1.10)	(0.90, 1.08)	(0.89, 1.08)
RLD (R2)	Lab (PEG/ETOH)	(1.12, 1.32)	(1.12, 1.32)	(1.09, 1.31)	(1.09, 1.31)

**Table 3:** BE analysis of the differences in the log-transformed C_max_ and AUC_0–6h_ between products in the upper skin layers. The C_max_ and AUC_0–6h_ were extracted from the combined normalized SRS intensity–time profiles. The C_max_ and AUC_0–6h_ of four ROIs and three depth slices (0–16 µm) from a sample were averaged into one value before BE analysis. The CI was calculated using the regular average BE (ABE) calculation method mentioned in the FDA guidance for IVPT studies. The output CIs were converted back to non-log-transformed values and written as (low limit, high limit).

Product 1	Product 2	90% Confidence Interval for the Mean Difference in the C_max_ Values (Product 2, Product 1)		90% Confidence Interval for the Mean Difference in the AUC_0–6h_ Values (Product 2, Product 1)	
		Lipid-Rich Intralamellar Region	Lipid-Poor Corneocyte Region	Lipid-Rich Intralamellar Region	Lipid-Poor Corneocyte Region
RLD (R1)	RLD (R2)	(0.91, 1.02)	(0.92, 1.03)	(0.93, 1.07)	(0.93, 1.07)
RLD (R1)	Generic	(0.93, 1.03)	(0.92, 1.02)	(0.92, 1.04)	(0.91, 1.03)
RLD (R1)	Lab (PEG/ETOH)	(1.08, 1.24)	(1.09, 1.24)	(1.08, 1.27)	(1.08, 1.28)
RLD (R2)	Generic	(0.95, 1.08)	(0.94, 1.07)	(0.92, 1.05)	(0.91, 1.04)
RLD (R2)	Lab (PEG/ETOH)	(1.11, 1.29)	(1.11, 1.29)	(1.08, 1.28)	(1.08, 1.28)

## Data Availability

The raw data supporting the conclusions of this article will be made available by the authors on request.

## Supplementary Materials

The following supporting information can be downloaded at https://www.mdpi.com/article/10.3390/pharmaceutics17091193/s1: Figure S1: Flowchart depicting implementation of BE study of topical products using SRS imaging. Figure S2: SRS spectrum of tretinoin powder. Figure S3: SRS intensity of reference polymer films made with different concentrations of tretinoin. Figure S4: (A) Correlation between SRS and transmission intensities in 1% tretinoin polymer film positioned under skin. (B) Graphical illustration demonstrating normalization of raw SRS signal to reference transmission point. Figure S5: SRS intensity–time profiles. Figure S6: Steps to obtain raw SRS intensity–time profile, standard film normalized SRS intensity–time profile, and combined normalized SRS intensity–time profile. Figure S7: (A) Schematic representation of the laser beams focused at a specific depth in a skin sample. (B) Es-timation of the power of the transmitted light passing through a human skin sample with different thicknesses. Figure S8: Statistical power vs. sample size curve, the C_max_ and AUC_0–6h_ values were from “combined normalized SRS intensity–time pro-files” in lipid-poor corneocyte regions. Figure S9: Statistical power vs. sample size curve, the C_max_ and AUC_0–6h_ values were from “combined normalized SRS intensity–time profiles” in lipid-rich intralamellar regions. Table S1: List of ingredients, their chemical structures, and their concentrations in Retin-A^®^ 0.1% cream and tretinoin 0.1% cream. Reference [50] are cited in the supplementary materials.
