# Supplementary material for: Improved Dual-Modality Bioequivalence Evaluation of Topical Formulations Within Human Skin Using Stimulated Raman Scattering Microscopy
Source: Pharmaceutics. 2025 Sep 13;17(9):1193. doi: 10.3390/pharmaceutics17091193 (PMC12473907; doi:10.3390/pharmaceutics17091193)
Supplement: Supplementary file 1 [file pharmaceutics-17-01193-s001.zip › pharmaceutics-3781312-supplementary.pdf]

Article

Improved Dual-Modality Bioequivalence Evaluation of Topical Formulations Within Human Skin Using Stimulated Raman Scattering Microscopy

Dandan Tu [1], Nick-Sidney Lemberger [2], Kristin Wallmeier [2], Jackson Riseman [1], Benjamin A. Kuzma [1,†], Yuxiao Wei [1], Ting Chean Khoo [1], Elena Rantou [3], Priyanka Ghosh [4], Markham C. Luke [4], Sam G. Raney [4], Carsten Fallnich [2] and Conor L. Evans [1,*]

[1] Wellman Center for Photomedicine, Massachusetts General Hospital, Harvard Medical School, CNY149-3, 13th St, Charlestown, 02129, MA, USA; datu@mgh.harvard.edu (D.T.); ben_kuzma@vrtx.com (B.A.K.); tkhoo@mgh.harvard.edu (T.C.K.)

[2] Institute of Applied Physics, University of Münster, Münster 48149, Germany; fallnich@uni-muenster.de (C.F.)

[3] Office of Biostatistics, Office of Translational Sciences, Center for Drug Evaluation and Research, U.S. Food and Drug Administration, Silver Spring, 20993, MD, USA;

[4] Office of Research and Standards, Office of Generic Drugs, Center for Drug Evaluation and Research, U.S. Food and Drug Administration, Silver Spring, 20993, MD, USA;

* Correspondence: evans.conor@mgh.harvard.edu

† Current Address: Drug Metabolism and Pharmacokinetics, Vertex Pharmaceuticals, Boston, 02210, MA, USA.

1. Photostability of Tretinoin Formulations

Multiple experiments were carried out to assess the photostability of tretinoin formulations during repetitive SRS imaging. In one experiment, tretinoin powder was imaged from 1100 cm^{-1} to 1700 cm^{-1} with a step size of 4 cm^{-1}. SRS spectra were plotted using the raw SRS signal from tretinoin powder and subtracting the signal obtained from a region in the same field of view absent of sample. For a duration of 6 hours, the tretinoin powder was exposed to the laser continuously. Three spectra at 0 hours, 3.5 hours, and 6.5 hours of exposure were plotted. Peak positions and intensities in these spectra (0 hours, 3.5 hours, and 6.5 hours) were compared to evaluate tretinoin stability. In another experiment, two samples of 0.1 % tretinoin dissolved in dimethylsulfoxide (DMSO, from Sigma-Aldrich ,Saint Louis, MO, USA) were used. One sample was used to collect 35 repetitive SRS images: the first image was taken at 0 hours, and the final image was taken at a time point over 7 hours later. The other sample was only exposed to the laser for the collection of two SRS images: one image was collected at 0 hours, and the other image was collected at a point over 7 hours later. This latter sample was not exposed to the laser between the two SRS image collection time points. The ratio of SRS intensity values collected in "repetitive measurements" to "only two measurements" at the first time point was calculated. Similarly, the ratio of SRS intensity values collected in these two samples at the final time point was calculated. These two ratio values were compared in order to confirm tretinoin photostability. In another experiment, the amount of tretinoin in the topical products after imaging and the topical product without exposure to the lasers were compared. The topical products Retin-A® 0.1% cream (Bausch Health US, LLC, Bridgewater, NJ, USA) and Tretinoin 0.1 % cream (Padagis US, LLC, Minneapolis, MN, USA) were put between two glass coverslips and imaged for over 7 hours using the SRS microscope. After imaging, the creams were then scraped off the glass and dissolved in DMSO. The two creams that were not exposed to lasers were dissolved in DMSO. An Agilent 1260 LC system (Agilent, Santa Clara, CA, USA) equipped with a triple-quad mass spectrometer was used to quantify the tretinoin in these samples. The following parameters were used in the measurement: C18 column (2.1 × 50mm, 1.8 μm); 2 μL injection volume; solvents used were

Acetonitrile as solution A and 10 mM ammonium acetate in water as solution B; gradient used was 30% to 100% A in 4 minutes, then 100% A for 2 minutes; positive ionization mode, MRM transition 301 → 283; retention time: 2.4 minutes.

2. Workflow in Bioequivalence Study of Tretinoin Topical Products

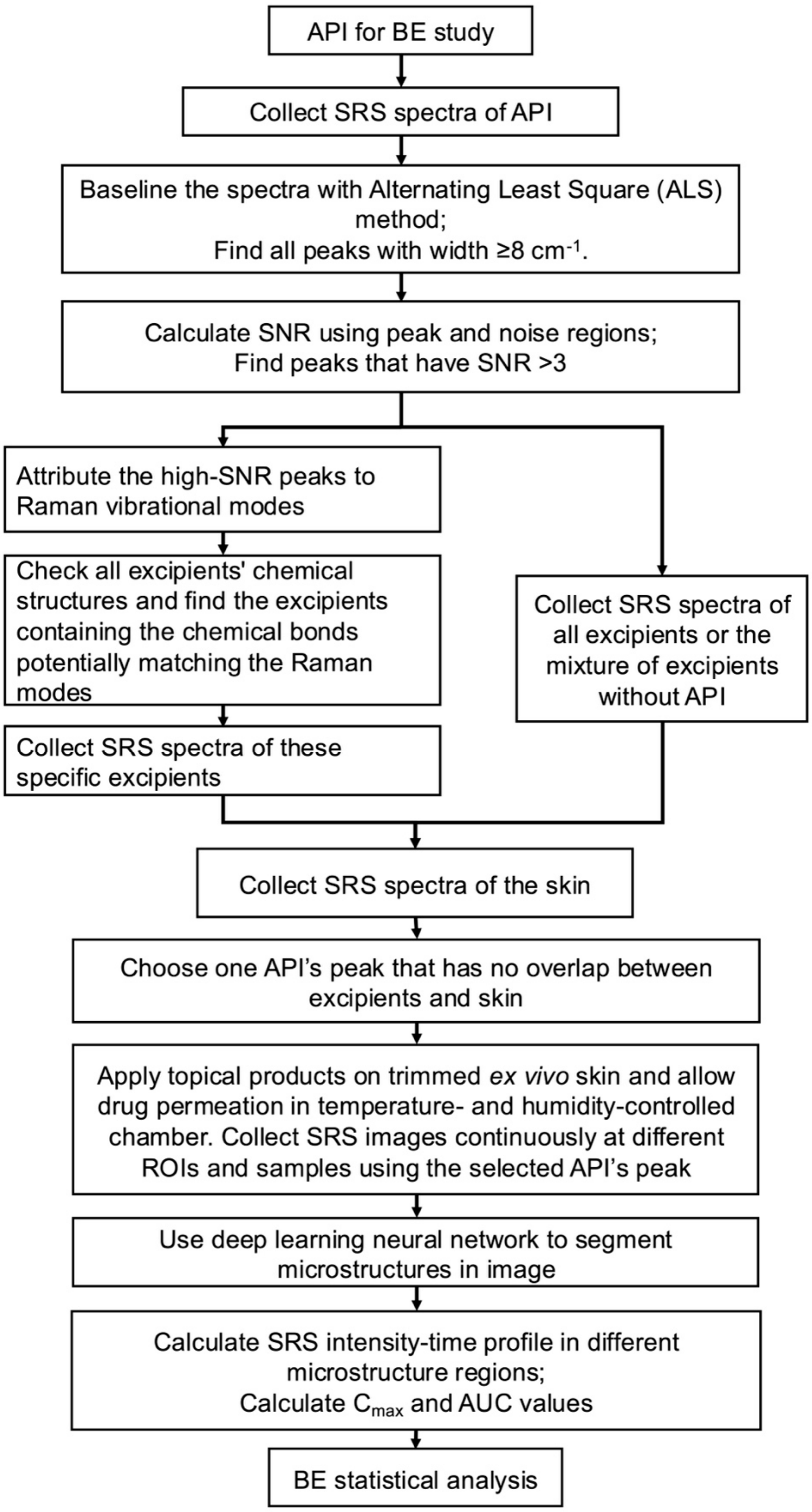

Figure S1. Flowchart depicting implementation of BE study of topical products using SRS imaging.

3. Tretinoin SRS Spectra

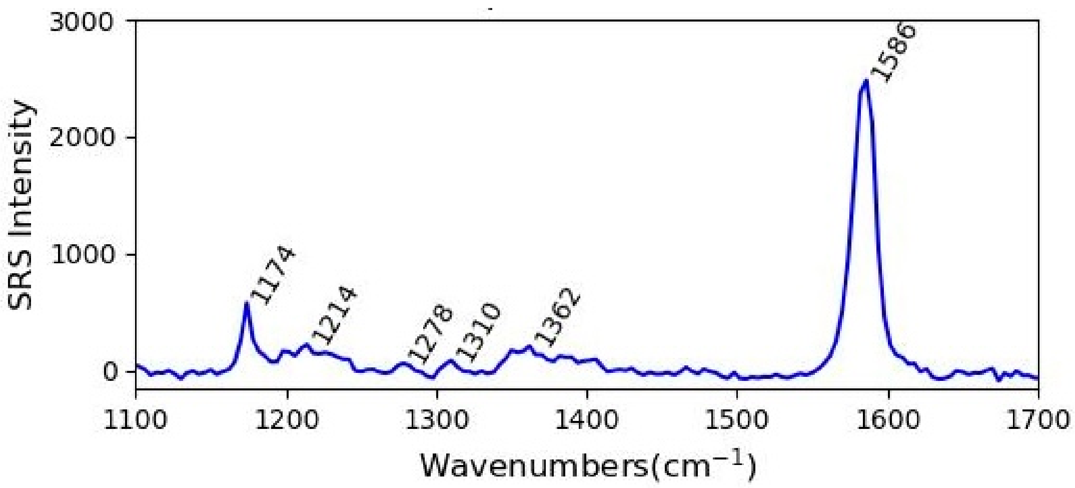

Figure S2. SRS spectrum of tretinoin powder. The SRS spectrum was plotted using raw SRS spectra from tretinoin powder after subtracting the air background. It was then baselined with the Alternating Least Square (ALS) method. All peaks with width ≥ 8 cm^{-1} and SNR > 3 were labeled.

4. Ingredients of Tretinoin Topical Products

Table S1. List of ingredients, their chemical structures, and their concentrations in Retin-A® 0.1% cream and tretinoin 0.1% cream

Ingredient	Concentration	Chemical Structure
Tretinoin (all-trans-retinoic acid)	0.1% w/w	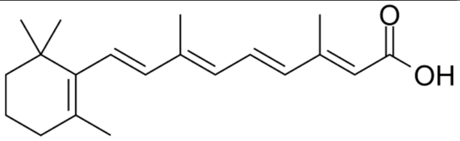
Butylated hydroxytoluene	≤ 0.8% according to safety standards	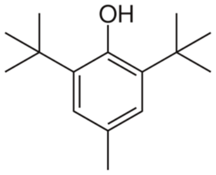
Sorbic acid	-	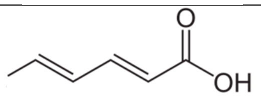
Stearic acid	-	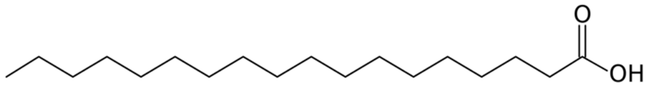
Isopropyl myristate	-	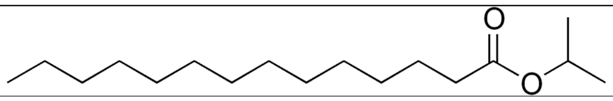
Polyoxyl 40 stearate	-	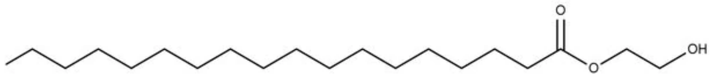
Stearyl alcohol	-	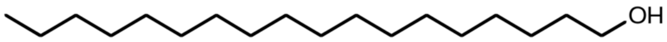

Xanthan gum	-	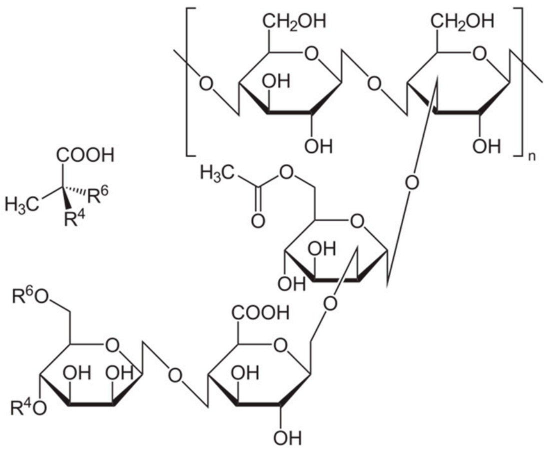

5. Tretinoin Reference Polymer Film

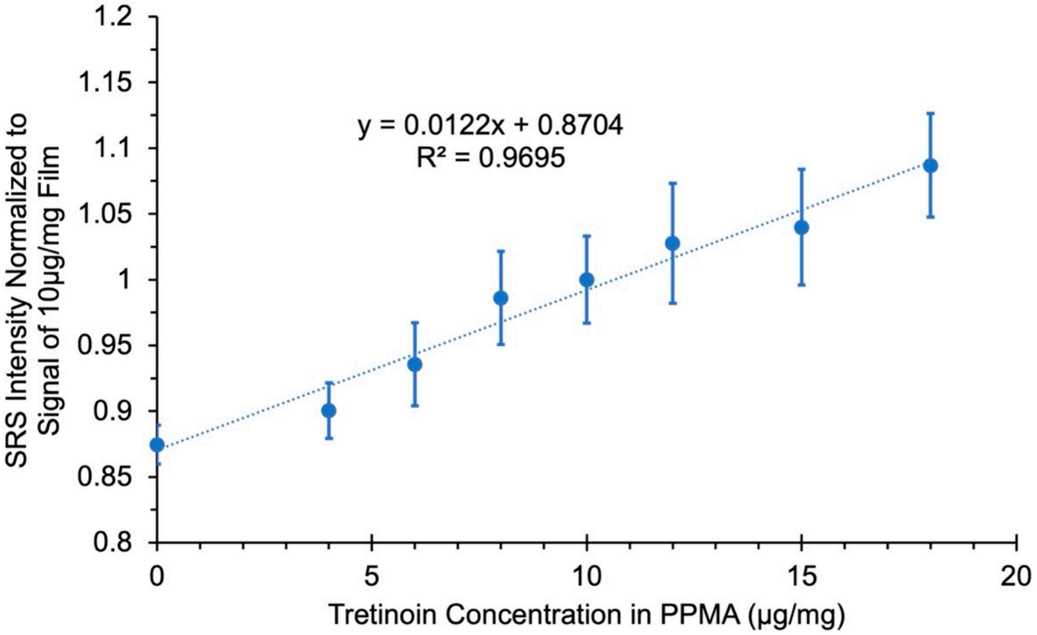

Figure S3. SRS intensity of reference polymer films made with different concentrations of tretinoin. The y-axis values are SRS intensity measured at 1586 cm^{-1}.

6. Correction Formula for Variance in Skin Thicknesses

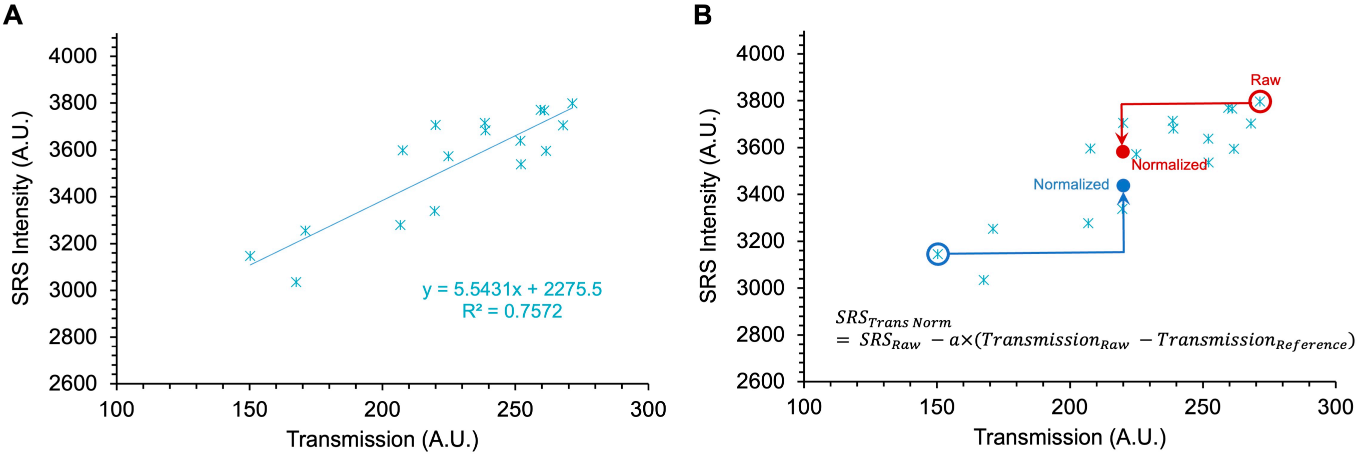

Figure S4. (**A**) Correlation between SRS and transmission intensities in 1% tretinoin polymer film positioned under skin. The skin samples were from a 55-year-old female, and their thicknesses were within 0.329 mm - 0.535 mm, with an average value of 0.416 mm. (**B**) Graphical illustration demonstrating normalization of raw SRS signal to reference transmission point. The circles indicate the original data points. The solid circles show the points after normalization using $SRS_{TransNorm} = SRS_{Raw} - a \times (Transmission_{Raw} - b)$. 'a' represents a factor to scale the transmission changes, and 'b' represents the reference transmission point ($Transmission_{Reference}$). The normalized points in this plot use an 'a' value of 4.2 and a 'b' value of 220.

7. Intensity–Time Profile of the Drug

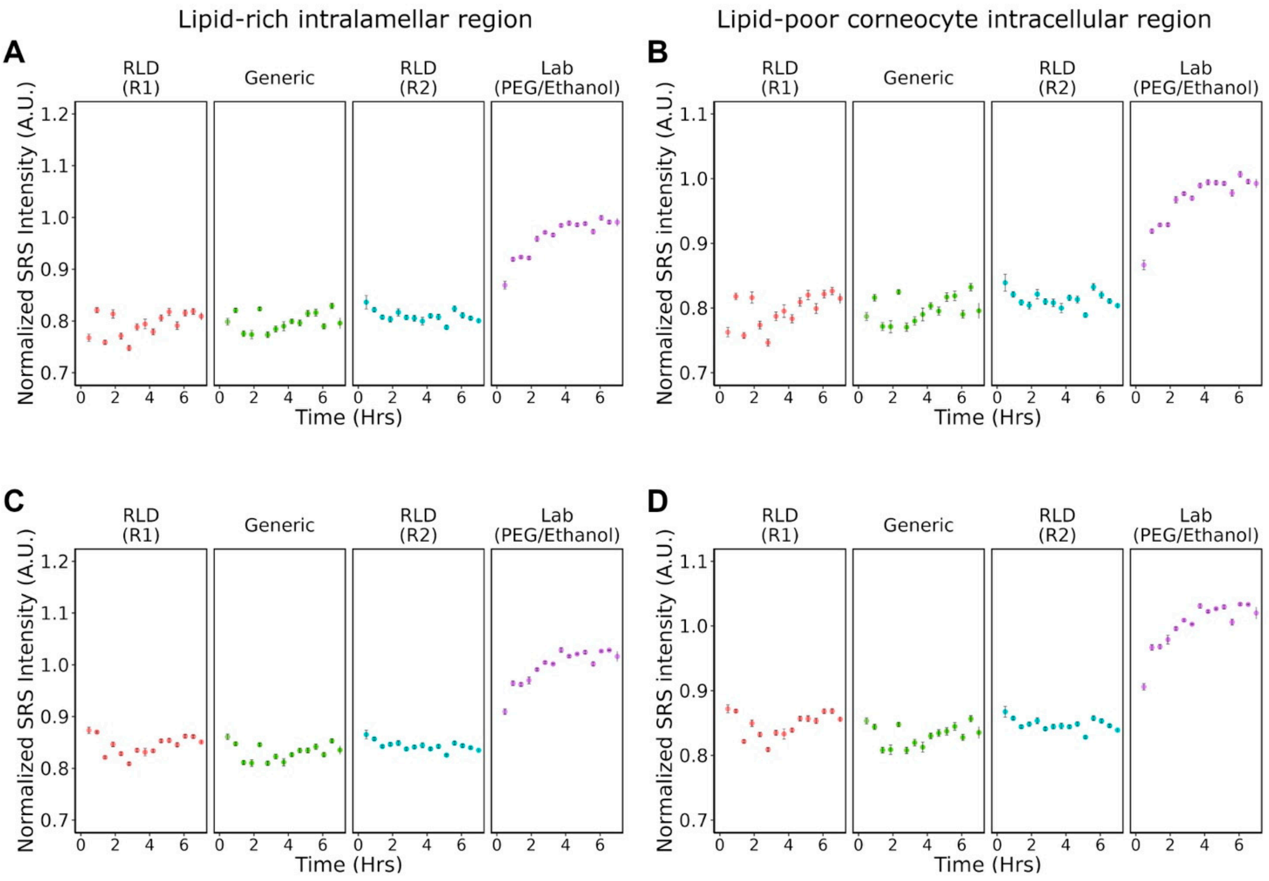

Figure S5. SRS intensity–time profiles. (**A-B**) Averaged standard film normalized SRS intensity–time profiles, and (**C-D**) averaged standard film and pixel-wise transmission-normalized SRS intensity–time profile, showing the uptake of tretinoin in the lipid-rich intralamellar region and the lipid-poor corneocyte intracellular region. Reference product (R1 and R2): Retin-A® 0.1% cream; generic product: Tretinoin 0.1 % cream; lab formulations: 0.1% w/w tretinoin in ethanol and PEG-400. Skin layers: 0-16μm. The error bar shows the standard error of the mean (SEM) of all ROIs of samples of each treatment group. Each group includes data from three depth slices of three ROIs on ≥4 independent abdominal skin samples from each of the 4 skin donors.

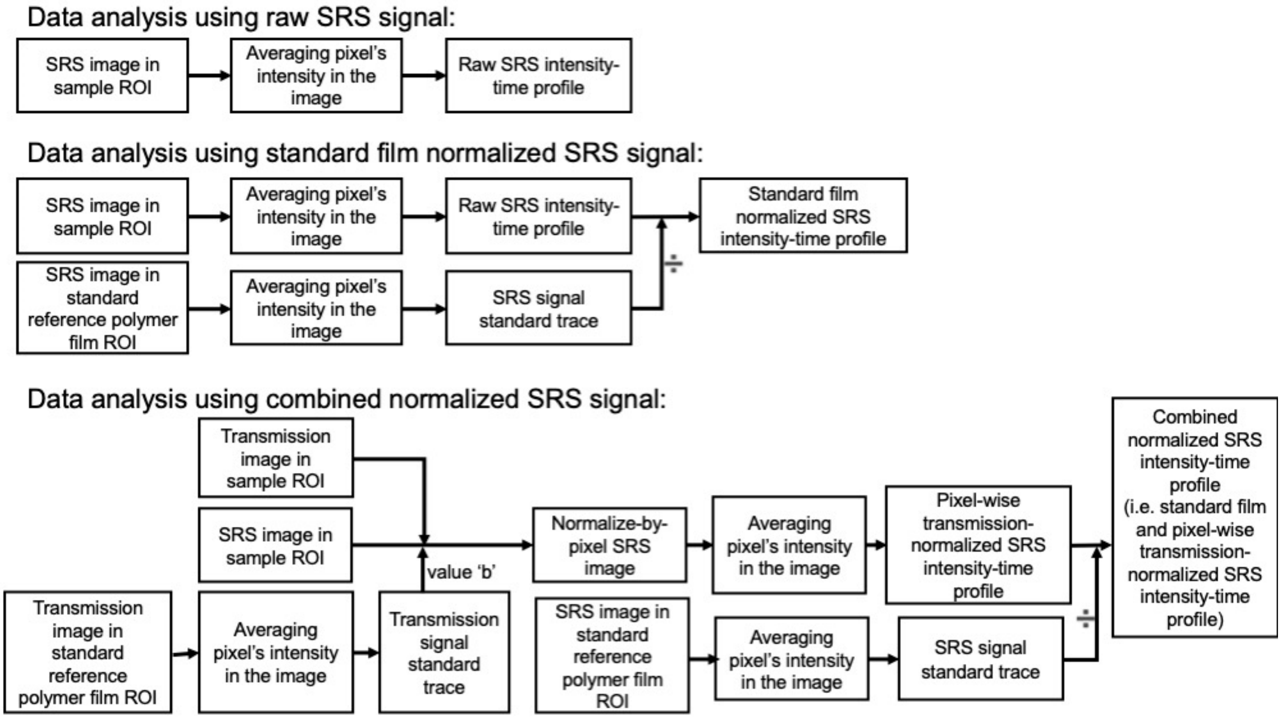

Figure S6. Steps to obtain raw SRS intensity–time profile, standard film normalized SRS intensity–time profile, and combined normalized SRS intensity–time profile. The division sign "÷" in the figure refers to the division of the intensity-time profile by the standard trace.

8. Estimation of Power Loss at Different Skin Depths

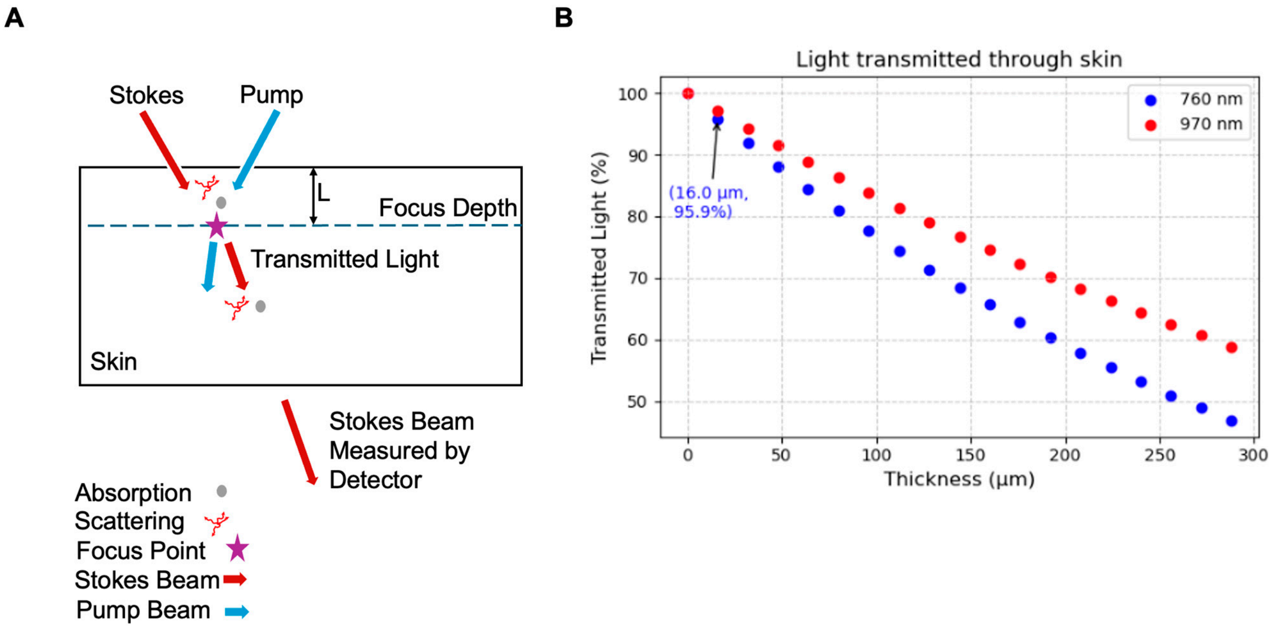

Figure S7. (**A**) Schematic representation of the laser beams focused at a specific depth in a skin sample. (**B**) Estimation of the power of the transmitted light passing through a human skin sample with different thicknesses.

The generation of the SRS signal involves the interaction between the pump beam and the Stokes beam at the focal point. As shown in Figure S7A, pre-focal scattering/absorption on the pump beam and the Stokes beam would affect the generation of the SRS signal. Because the detector only detects the Stokes beam, the power loss on the pump beam before the focus point cannot be accounted for. The higher power loss on the pump

beam, the worse performance in normalizing SRS readout variance using transmission. A light attenuation calculation is used to determine the conditions when the power loss in the pump beam is low.

In our SRS microscope, when imaging at the wavenumber of 1586 cm^{-1}, the pump beam has a wavelength of around 897 nm, and the Stokes beam has a wavelength of around 1046 nm. When a light passes through tissue, the transmitted light intensity can be estimated using $I_{transmitted} = I_{in} \times e^{-(\mu_s' + \mu_a)L}$. μ_s' is the reduced scattering coefficient and μ_a is the absorption coefficient. I_{in} is the input intensity and $I_{transmitted}$ is the intensity at a depth of L. For ex vivo abdominal skin with Fitzpatrick skin type I-II, μ_s' value can be set as 2.45 mm^{-1} when the wavelength is 760 nm and 1.8 mm^{-1} when the wavelength is 970 nm. The μ_a value can be set as 0.18 mm^{-1} when the wavelength is 760 nm and 0.038 mm^{-1} when the wavelength is 970 nm [50]. The estimated transmitted light at different depths of skin is shown in Figure S7B. As shown in Figure S7B, the estimated transmitted light power is above 95% when passing through 16 µm skin. This indicates a low signal loss before the focus if imaging at 16 µm. When passing through more skin, the power loss keeps increasing and the impact from the pump beam power loss would become more significant. Based on this, the developed transmission normalization method was restricted to an SRS imaging depth of 0–16 µm, where light power loss is minimal.

9. Statistical Power Analysis for Comparison of PK Parameters

The RLD and the lab-made formulation are expected to have differences in drug permeation. Statistical power analysis was used to determine the minimal sample size needed to detect the difference on the PK parameters (C_{max} and $AUC_{0\text{–}6h}$) when comparing the RLD treatment group (R1 and R2) and the lab-made formulation group (Lab). According to the Shapiro–Wilk normality test, the C_{max} and $AUC_{0\text{–}6h}$ values of the RLD treatment group (R1 and R2) and the lab-made formulation group (Lab) were normally distributed. Next, t-test power analysis was used to calculate the power values with different numbers of samples per treatment group. The results are shown in Figures S8 and S9.

As shown in Figure S8, a minimum of 9 to 13 samples is needed to reach the 80% power for comparing the PK parameters. A similar observation is shown in Figure S9.

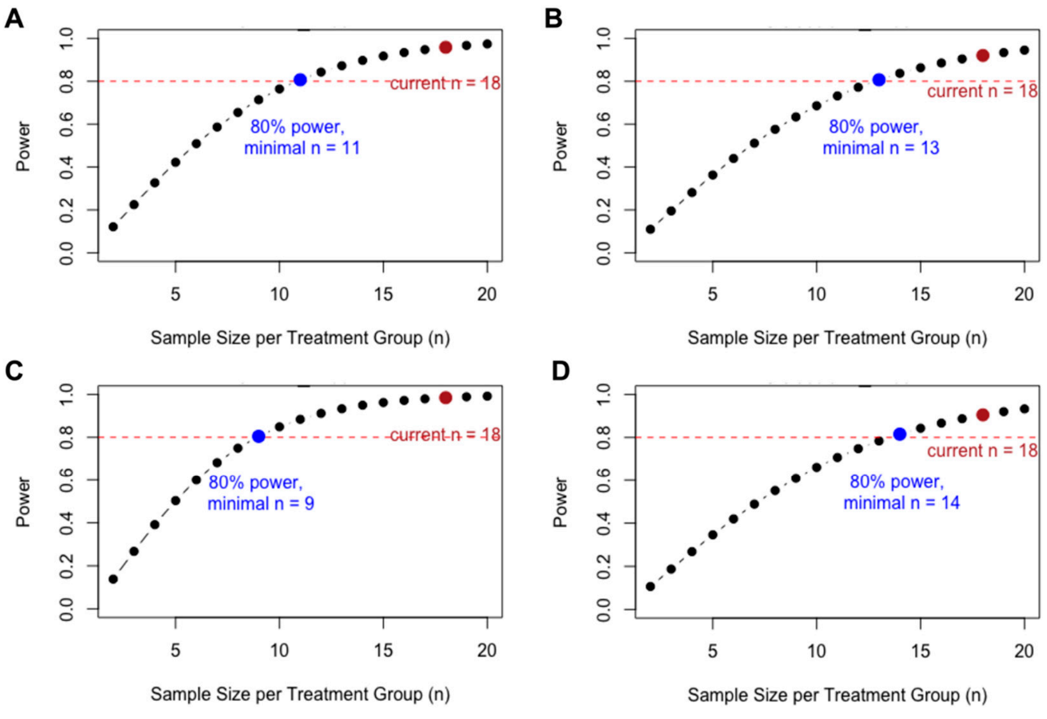

Figure S8. Statistical power vs. sample size curve for (**A**) comparing the C_{max} values from R1 group and Lab group, (**B**) comparing the $AUC_{0–6h}$ values from R1 group and Lab group, (**C**) comparing the C_{max} values from R2 group and Lab group, and (**D**) comparing the $AUC_{0–6h}$ values from R2 group and Lab group. The C_{max} and $AUC_{0–6h}$ values were from "combined normalized SRS intensity–time profiles" in lipid-poor corneocyte regions.

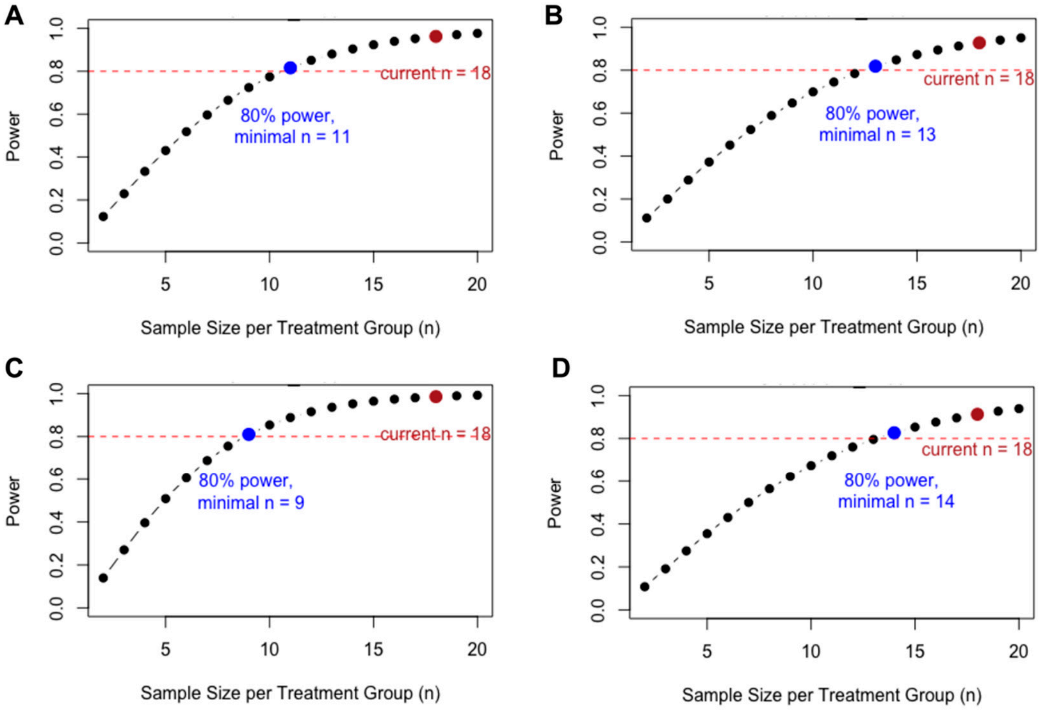

Figure S9. Statistical power vs. sample size curve for (**A**) comparing the C_{max} values from R1 group and Lab group, (**B**) comparing the $AUC_{0–6h}$ values from R1 group and Lab group, (**C**) comparing the C_{max} values from R2 group and Lab group, and (**D**) comparing the $AUC_{0–6h}$ values from R2 group and Lab group. The C_{max} and $AUC_{0–6h}$ values were from "combined normalized SRS intensity–time profiles" in lipid-rich intralamellar regions.
